# Sexual conflict as a constraint on asexual reproduction: an empirical review

**DOI:** 10.1111/brv.70064

**Published:** 2025-08-11

**Authors:** Daniela Wilner, Russell Bonduriansky, Nathan W. Burke

**Affiliations:** ^1^ Evolution and Ecology Research Centre, School of Biological, Earth and Environmental Sciences, The University of New South Wales, UNSW Sydney NSW 2052 Australia; ^2^ Institute of Cell and Systems Biology of Animals, Department of Biology Universität Hamburg Mittelweg 177 Hamburg 20148 Germany

**Keywords:** facultative parthenogenesis, paradox of sex, evolution of parthenogenesis, sexual conflict, reproductive modes, asexuality, costs of sex, animals, invertebrates, behaviour

## Abstract

Theory predicts that facultatively asexual animals, which can leverage the advantages of both sexual and asexual reproduction, should outcompete obligately sexual and obligately asexual animals. Yet, paradoxically, obligate sexual reproduction predominates in many animal lineages, while the most flexible form of facultative asexuality (i.e. facultative parthenogenesis) appears to be rare. Recent theoretical work suggests that sexual conflict could help to resolve this paradox. Males that coercively fertilise females' eggs may, in the process, prevent alleles for parthenogenesis from spreading by limiting opportunities for asexual reproduction. Coercive males may also inhibit asexual reproduction by making resistance to sex disproportionately costly for females. In this review, we outline evidence of interactions with males that could impose costs on parthenogenetic females or hinder their ability to reproduce parthenogenetically in diverse animal taxa. The evidence suggests that such interactions between the sexes have the potential to mediate sexual conflict over mating and reproductive mode, both within facultative species and between closely related sexual and asexual taxa. However, the relative costs of sex and parthenogenesis are clearly context dependent, and much remains unknown. The most direct evidence for male inhibition of parthenogenesis comes from stick insects, but several other systems offer promising avenues for further investigation. Further research on the costs of mating and resistance in such systems could shed light on the reasons for the puzzling rarity of facultative parthenogenesis in nature.

## I. INTRODUCTION

The relative abundance of obligately sexual animals has troubled evolutionary biologists for decades because, despite its hypothesised capacity to promote purifying selection and adaptation, sexual reproduction can be very costly in the short term (Williams, 1975; Maynard Smith, 1978; Bell, 1982; Rankin, Dieckmann & Kokko, 2011; Lehtonen, Jennions & Kokko, 2012; Arnqvist & Rowe, 2005). However, theory suggests that obligate asexuality has its own problems in the long term. Due to the lack of recombination between the genotypes of different individuals, and often low genetic diversity, asexual populations should be less able to adapt quickly to changing environments (Maynard Smith, 1978), and they could be more susceptible to the accumulation of deleterious mutations (Muller, 1964). Some types of asexual reproduction can also lead to the loss of heterozygosity (Suomalainen, 1950), making some asexuals more susceptible to expressing recessive deleterious traits. Both obligately sexual and obligately asexual reproductive strategies therefore seem paradoxical (Burke & Bonduriansky, 2017).

Theory suggests that the optimal strategy is a flexible one: switching between sexual and asexual reproduction so as to minimise the costs and maximise the benefits of each. Models suggest that animals that engage in occasional sex between bouts of asexual reproduction could gain all the benefits of sex while avoiding most of the costs (D'Souza & Michiels, 2010; Hurst & Peck, 1996), and that such animals should outcompete both their obligately sexual and obligately asexual counterparts (Burke & Bonduriansky, 2017). Varying levels of reproductive flexibility have evolved in several animal lineages (Bell, 1982), but perhaps the most flexible form is facultative parthenogenesis. In facultatively parthenogenetic systems, each female is capable of both sexual reproduction (i.e. producing sons and daughters from eggs that have been fertilised by a male) and parthenogenesis (i.e. producing daughters from unfertilised eggs), without the need for any environmental trigger to switch between the two reproductive modes (as opposed to cyclical parthenogens, where the switch is generational and occurs in response to specific environmental cues).

While theory suggests that flexible strategies that incorporate both sexual and asexual reproduction (particularly facultative parthenogenesis) should outcompete obligately sexual and obligately asexual strategies (D'Souza & Michiels, 2010; Hurst & Peck, 1996; Pamilo, Nei & Li, 1987; Burke & Bonduriansky, 2017), animal species abundances in nature do not match this prediction, and obligate sex appears to be the norm in many animal lineages (Bell, 1982; Vrijenhoek *et al*., 1989). It has been suggested that physiological constraints might hinder the evolution of parthenogenesis (Engelstädter, 2008; Galis & Alphen, 2020). Indeed, certain aspects of development may strongly constrain the evolution of parthenogenesis in some animals; for example, unfertilised mammal eggs cannot develop naturally due to genomic imprinting (Kono, 2006). However, other hypothesised constraints have been overcome or circumvented repeatedly and in diverse ways across metazoans; for example, the ability to produce unfertilised eggs with appropriate ploidy levels has evolved many times, *via* a great diversity of mechanisms (Suomalainen, Saura & Lokki, 1987; Simon *et al*., 2003). Additionally, reports of occasional parthenogenetic reproduction (tychoparthenogenesis) across taxa (e.g. Chapman *et al*., 2007; Markow, 2013; Booth *et al*., 2023) suggest that the potential for parthenogenesis is present in many animal lineages where reproduction typically occurs *via* sex (Normark & Kirkendall, 2009), and that physiological constraints do not provide a complete explanation for the rarity of facultative parthenogenesis. The role of physiological constraints is especially unclear in taxa where parthenogenesis has evolved repeatedly (Simon *et al*., 2003). What else is hindering the invasion of obligately sexual populations by mutants capable of parthenogenetic reproduction?

**Fig. 1:**
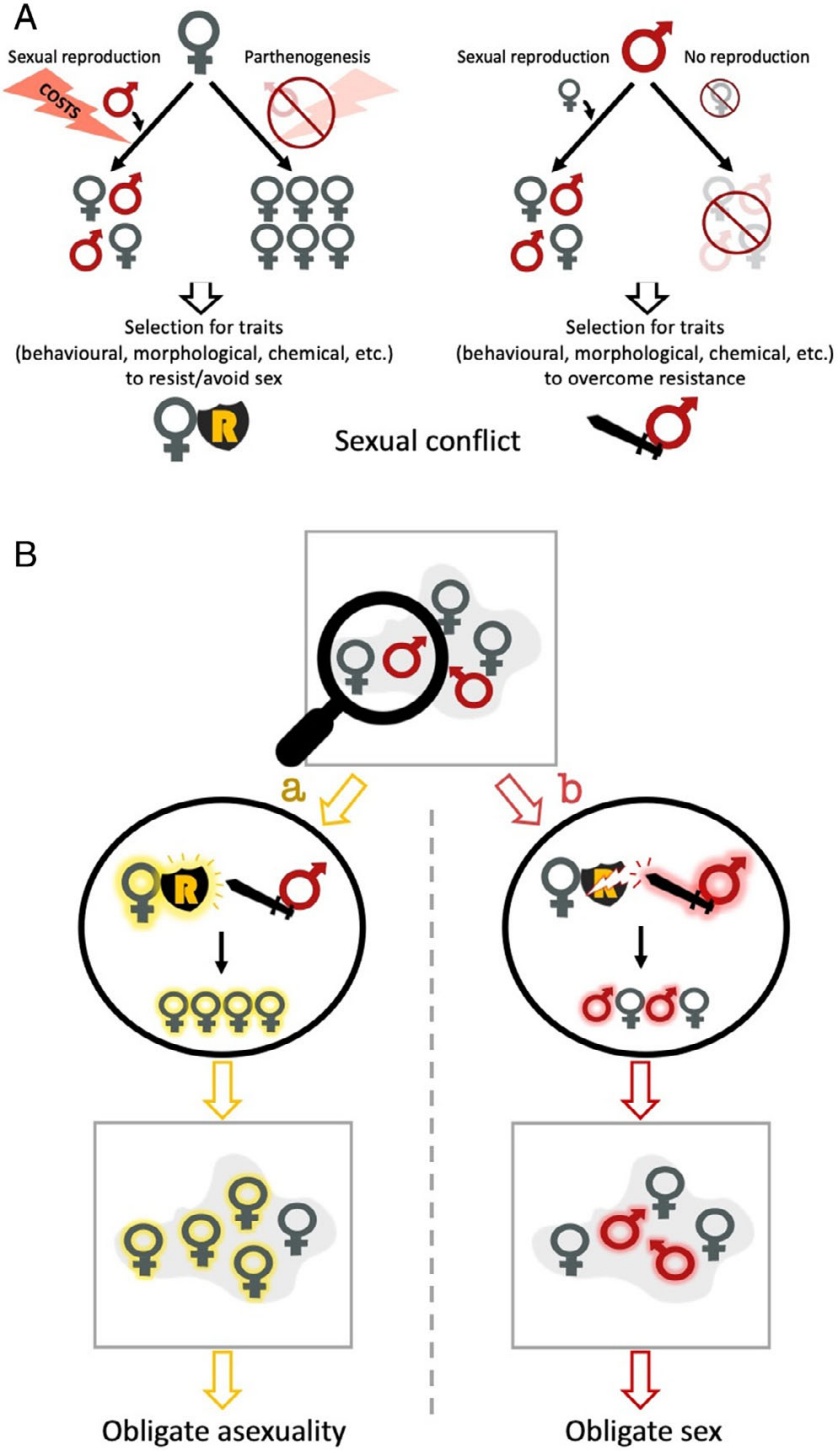
Graphical representation of the sexual conflict hypothesis [incorporating ideas from Burke & Bonduriansky (2017), Kawatsu (2013a), Burke & Bonduriansky (2018b) and van der Kooi & Schwander (2014)]. (A) Since sex can impose direct and indirect costs on females, those that can reproduce parthenogenetically may be under selection to avoid or resist sex. Since males can only reproduce *via* sex, they may be under selection to overcome female avoidance or resistance. The conflicting selection pressures on males and females can cause the evolution of sexually antagonistic traits, here represented by a shield (female resistance) and a sword (male persistence or coercion). (B) The ensuing evolutionary arms race may lead to the loss of facultative parthenogenesis in a population. If females evolve effective resistance and only reproduce parthenogenetically (outcome ‘a’, left), males could go extinct because parthenogenetic eggs only produce daughters, rendering the population effectively asexual. Additionally, after a population is devoid of males, females' capacity for sexual reproduction may be lost *via* vestigialisation over the generations, rendering the females obligately asexual. Alternatively, if male coercion effectively enforces sexual reproduction (outcome ‘b’, right), males will continue to be produced in the population, and eventually the capacity for parthenogenesis may be lost, through vestigialisation or through parthenogenetic females suffering greater costs than sexual females (see Section I), rendering the population obligately sexual.

A possible explanation for the maintenance of obligate sexuality in animals is sexual conflict (Kawatsu, 2013a; Burke & Bonduriansky, 2017). When a facultatively parthenogenetic mutant arises in a sexual population, there could be intense sexual conflict over mating (Burke & Bonduriansky, 2017; see Fig. 1A) because males still need to mate to reproduce, while facultative females do not, and mating can be costly [e.g. due to male harm, sexually transmitted diseases, etc. (Daly, 1978; Arnqvist & Nilsson, 2000)]. Conflict over mating can cause selection for sexually antagonistic traits, such as female resistance to mating and male coercive behaviour (Parker, 1979; Arnqvist & Rowe, 2005). If females gain the upper hand in the resulting arms race, males could go extinct, resulting in all‐female populations (Burke & Bonduriansky, 2018b). Conversely, if males gain the upper hand in the arms race, the ability of parthenogenetic females to reproduce asexually could be limited, potentially leading to the complete extinction of parthenogenesis (Burke & Bonduriansky, 2017; Kawatsu, 2013a; see Fig. 1B). Thus, depending on the relative efficacy of male coercion *versus* female resistance, sexual conflict could drive the spread or suppression of parthenogenetic strategies (Kawatsu, 2015, 2013a; Burke & Bonduriansky, 2017, 2019b). In this way, sexual conflict may cause facultative parthenogenesis to be unstable and often lost; we henceforth refer to this idea as the sexual conflict hypothesis (Fig. 1).

Males could limit the evolution of parthenogenesis in several ways. If females very rarely get the chance to reproduce asexually, selection to maintain the required physiology will be weak, and it may be subject to vestigialisation (decay *via* drift). In addition, since facultative parthenogenesis is a type of plasticity, the required physiological systems are likely to be costly or subject to trade‐offs (DeWitt, Sih & Wilson, 1998). If the chance to use this plasticity rarely arises, the benefit of having it is unlikely to outweigh the cost of maintaining it, and it may be selected against. Furthermore, investing in resistance could limit facultative females' ability to reproduce if the costs of resistance are especially high. In such a scenario, it may be more convenient to mate than to resist (Gerber & Kokko, 2016).

Sexual conflict might not prevent the invasion of facultatively parthenogenetic mutants into obligately sexual populations if females avoid males entirely (e.g. occupy male‐free habitat patches; Burke & Bonduriansky, 2019b). Additionally, post‐copulatory processes may prevent coercive matings from resulting in fertilisation. In some facultatively parthenogenetic species, females can lay unfertilised eggs even after mating (e.g. Wilner *et al*., 2025; Morgan‐Richards, Langton‐Myers & Trewick, 2019). However, sexual conflict could still hinder the spread of parthenogenesis in species where mating does not always result in fertilisation. In fact, female resistance to fertilisation could exacerbate the process.

Parthenogenetic females can be subject to the costs of mating and resistance (e.g. energetic costs, injury, etc.) regardless of whether or not fertilisation occurs. They may even suffer greater costs from these interactions than their sexual counterparts (Kawatsu, 2013b; Dagg, 2006). For example, if parthenogenetic females exhibit more resistance behaviours than sexual females do, and if these behaviours are costly (e.g. energetically or due to risk of injury), exposure to males could reduce the fitness of parthenogenetic females more than that of sexual females. Additionally, parthenogens that have been isolated from males for many generations (i.e. in all‐female populations, such as those found in facultatively parthenogenetic stick insects; Miller *et al*., 2024a) may be less adapted to withstand harm caused by males (Kawatsu, 2013b). Thus, sexual conflict could disproportionately reduce the fitness of parthenogenetic females, potentially giving obligately sexual females a fitness advantage.

Furthermore, sexual conflict over fertilisation can lead to the evolution of sexually antagonistic post‐copulatory traits (reviewed in Firman *et al*., 2017; Firman, 2018; Arnqvist & Rowe, 2005) that may impose costs on any females that mate (e.g. male seminal fluids can decrease female survival in *Drosophila*; Chapman *et al*., 1995). If parthenogenetic females do not gain counterbalancing benefits of sex after mating or resisting, and sexual females do (e.g. through ‘sexy sons’), the net cost of mating could be disproportionately high for parthenogenetic females. If mating cannot be avoided, females may then be under selection to minimise net costs by foregoing resistance to fertilisation and reproducing sexually (Kawatsu, 2013b, 2015).

Selection may or may not favour parthenogenetic reproduction when sexual reproduction is an option. For example, sexual reproduction may be beneficial for females if it increases the genotypic diversity or viability of their offspring (Hadany & Otto, 2007). Moreover, it is likely that the relative benefits of parthenogenesis and sex are context dependent, just as sexual conflict is often environment dependent in obligately sexual animals (Bonduriansky, 2014; Fricke *et al*., 2009; Fricke, Bretman & Chapman, 2010). Thus, parthenogenetic reproduction might be favoured in some contexts but not others, and the presence, magnitude, and outcomes of sexual conflict may be very variable. The rigorous study of natural populations of facultative parthenogens is essential to evaluate the sexual conflict hypothesis.

Extensive modelling has investigated the effects of sexual conflict on the evolution of parthenogenesis (Burke & Bonduriansky, 2018b, 2017, 2019b; Gerber & Kokko, 2016; Kawatsu, 2015, 2013a,b), but empirical work on this question is sparse. This is partly due to the rarity of facultative parthenogens (and dearth of facultative parthenogens among laboratory model animals), and further hindered by the lack of basic life‐history research on such animals. Few cases of parthenogenesis are known in vertebrates, and although it appears to be much more common in invertebrates, the reproductive mode of many species is either unconfirmed or completely unknown. An additional difficulty may stem from the confusion caused by the sheer variety of reproductive modes in nature and the many disagreements over terminology in the literature (see Table 1 for a glossary of how key terms are used here). Researchers often disagree on how to define and classify the many ways in which animals reproduce, and even disagree on what constitutes sex – whether the defining feature is mating, syngamy/fertilisation, meiosis, or recombination (Fusco & Minelli, 2023; Lehtonen & Kokko, 2014). We here use sexual reproduction to mean the production of offspring that carry genetic contributions from a female and a male, *via* fertilisation.

**Table 1:** Glossary. Definitions of key terms. Note that this review is focused on gonochoristic, anisogamous animals (i.e. animals with two sexes residing in separate individuals, where males produce sperm and females produce eggs). While we have attempted to categorise the reproductive diversity of such animals, much variation occurs across a continuum from obligate sex to obligate asexuality, and the lines between categories are often blurred in reality (see Bell, 1982; Normark, 2003; Lehtonen *et al.*, 2013; Schwander & Oldroyd, 2016). Additionally, the reproductive modes and parthenogenetic capacities of many animals remain undescribed or poorly understood.

Term	Definition
Sexual reproduction	The production of offspring from fertilised eggs, bearing genomic contributions from a female (mother) and a male (father). Note that some authors advocate a broader definition that requires only the presence of recombination or re‐organisation of genetic material, and thereby encompasses parthenogenesis as a form of sexual reproduction.
Parthenogenesis	The production of offspring from unfertilised eggs. The term is here used in its narrowest sense, as a synonym for thelytoky (the production of *females* from unfertilised eggs), but other authors also use it to refer to arrhenotoky. Note that the term has been used in the literature to refer to both obligate and facultative parthenogenesis, as well as gynogenesis.
Thelytoky	The production of females from unfertilised eggs. (Note that many animals that reproduce *via* thelytoky are also capable of occasionally producing rare ‘spontaneous’ males, apparently due to errors in meiosis.)
Arrhenotoky	The production of males from unfertilised eggs.
Obligate parthenogenesis/obligate asexuality	An obligate (inflexible) strategy whereby only females exist, and these are only able to reproduce parthenogenetically. (Note that several putatively obligate asexual species are actually able to interbreed with males from closely related sexual species.)
Obligate sex	An obligate (inflexible) strategy whereby both males and females are only able to reproduce sexually.
Facultative parthenogenesis	Arguably the most flexible reproductive strategy, whereby every female in a population can reproduce either sexually (by mating with a male) or parthenogenetically (with no mating required), flexibly switching between the two reproductive modes, and in some cases using them simultaneously (i.e. mating but producing offspring from both fertilised and unfertilised eggs). Note that many authors use this term also to refer to cyclical parthenogenesis and tychoparthenogenesis.
Cyclical parthenogenesis	A somewhat flexible strategy in which sexually and asexually reproducing generations alternate, with the switch typically triggered by specific environmental cues (e.g. in annual cycles based on the seasons). Note that some authors refer to cyclically parthenogenic lineages as ‘sexual’ when comparing them to obligately asexual lineages.
Tychoparthenogenesis	The occasional production of offspring from unfertilised eggs by mutant females, in populations where the majority of females reproduce sexually and appear to be incapable of parthenogenesis – also known as spontaneous parthenogenesis.
Gynogenesis/pseudogamy	Systems where females produce offspring without genetic contribution from a father, but still need to mate to do so (typically acting as ‘sexual parasites’ of a closely related, obligately sexual species) – also known as sperm‐dependent parthenogenesis.
Androgenesis	Systems where males behave as sexual parasites: eggs and sperm fuse, but the nuclear genome of the egg is discarded, so offspring only inherit nuclear genes from their father.
Hybridogenesis	When interbreeding between different species results in fertile, sexually produced hybrid offspring, but grand‐offspring do not inherit any genes from one of the grandparents. For example, in *Bacillus* stick‐insects, a hybridogenic female only passes on her mother's genes to her own offspring, excluding those of her father. In other words, sexual reproduction occurs, but only one parent's genome is preserved in the germline through the generations – also known as hemiclonal reproduction.

We use a narrow definition of parthenogenesis (i.e. as a synonym of thelytoky), where mating is not necessary for female reproduction, and unfertilised eggs typically develop into daughters (although rare asexually produced males can also occur through errors in meiosis; e.g. Schwander *et al*., 2013). Several other systems with varying forms and degrees of asexuality exist (Normark, 2003; Bell, 1982), such as arrhenotoky (where unfertilised eggs produce males instead of females – e.g. in haplodiploid social insects), cyclical parthenogenesis (where some generations reproduce asexually, and others do so *via* mating and fertilisation, and the production of males and eggs requiring fertilisation is triggered by environmental change), and several systems where mating takes place but only one parent's genome is passed on to later generations (e.g. gynogenesis, hybridogenesis, and androgenesis; see Lehtonen *et al*., 2013). We will focus our review on thelytokous facultative parthenogenesis because the potential role of sexual conflict is especially clear in this reproductive system.

There is also vast diversity within the umbrella of thelytokous parthenogenesis. Firstly, there are many different mechanisms to produce eggs that can develop without being fertilised. Broadly, these are grouped into automixis (where meiosis occurs but diploidy of eggs is restored in some way) and apomixis (where diploid eggs are essentially produced by mitosis), but there is variation within these categories, with diverse outcomes for genetic diversity (Suomalainen *et al*., 1987). Secondly there are varying degrees of flexibility in the reproductive mode of females capable of parthenogenesis (e.g. Larose *et al*., 2023), forming a continuum from obligate parthenogenesis (i.e. species entirely made up of females that cannot reproduce sexually, even if they encounter males of closely related species), through facultative parthenogenesis (where every female can reproduce sexually or parthenogenetically), to tychoparthenogenesis (also known as spontaneous parthenogenesis, where sexual reproduction is the rule, and very few females are able to reproduce asexually, typically with low rates of embryo viability).

Here, we consider what empirical evidence exists for a role of sexual conflict in the establishment and maintenance of facultative parthenogenesis, and we highlight some promising systems to explore further. Studying intersexual interactions and their consequences for fitness in known facultative parthenogens can shed light on whether sexual conflict could halt the spread of facultative mutants in a sexual population, and help uncover which traits and interactions play key roles in this dynamic. Due to the very limited literature on male–female interactions in facultative parthenogens, we also examine interactions between closely related sexual and asexual lineages (e.g. species complexes where sister taxa differ in reproductive mode but overlap geographically). Conflict could also prevent the spread of obligate asexuality if costly interactions with sympatric males of closely related sexual species reduce the fitness of obligately asexual females. Further, we focus on systems where parthenogenesis is relatively common and successful, because intense sexual conflict over mating is not expected in tychoparthenogenetic systems, where developmental constraints often make reproductive output *via* parthenogenesis vastly inferior to sexual reproduction (Engelstädter, 2008). However, we do examine some clades where several species exhibit tychoparthenogenesis and there is information that is interesting to consider in the context of the sexual conflict hypothesis.

We do not review other systems that include elements of asexuality but are less relevant to the sexual conflict hypothesis. For example, the sexual conflict hypothesis does not apply in a straightforward way to systems where females always need to mate with a male to reproduce (e.g. gynogenesis) or to produce daughters (e.g. arrhenotoky or haplodiploidy). The sexual conflict hypothesis is also less straightforwardly applicable to eusocial insects (e.g. termites), or non‐thelytokous cyclical parthenogens like *Daphnia*, where females typically reproduce parthenogenetically but start producing asexually produced males and ‘resting eggs’ (which are resistant to extreme environments but require fertilisation) in response to certain environmental cues. While such systems fall outside the scope of this review, we would like to encourage further theoretical and empirical work on them in the context of the paradox of sex. Interesting work on sexual conflict is emerging in these systems (e.g. Lee, Solano Udina & Hansson, 2021).

For each taxon that we review, we ask whether there is empirical evidence of intersexual interactions that have the potential to affect negatively the fitness of females that reproduce parthenogenetically. The presence of sexual conflict alone does not demonstrate that sexual conflict inhibits the spread of facultative parthenogenesis. To constrain the evolution of parthenogenesis, sexual conflict would need to reduce the fitness of parthenogenetic females more than sexual females, or prevent them from reproducing parthenogenetically, or otherwise alter the relative costs of resistance, parthenogenesis, and sex in such a way that reproducing parthenogenetically becomes disadvantageous or rare. Investigating how and when sexual conflict affects parthenogenetic animals is an important first step in evaluating the sexual conflict hypothesis.

To determine whether there is sexual conflict in a facultatively parthenogenetic system, we need to compare female fitness when females do and do not interact with males, and when they do and do not mate (and ideally, when eggs are and are not fertilised). The most compelling evidence would be that fertilisation, mating, and/or interacting with males reduces female longevity or fecundity and/or offspring viability, quality, or reproductive success. All of these measures should be considered, as they may trade off with each other (for example, if mating harms females, but their sexually produced sons have very high fitness, sex may still be adaptive). However, studies thoroughly examining how males and sex affect various components of fitness in facultative parthenogens are rare, and those comparing the success of their sexually and asexually produced offspring even more so. Behavioural and morphological evidence can also be suggestive of sexual conflict, including male–female struggles, female avoidance of or resistance to mating, males injuring females during mating or mating struggles, or antagonistic coevolution in sexual morphology (e.g. specialised male structures to overcome female pregenital barriers).

All else being equal, if there is elevated sexual conflict in facultative parthenogens, facultatively parthenogenetic females should exhibit more sexually antagonistic traits (e.g. resistance behaviours or morphology) than their obligately sexual counterparts. Behavioural resistance should also differ in a context‐dependent way, as follows. If mating is more costly to fitness than not mating, facultatively parthenogenetic females should resist all mating (Burke & Bonduriansky, 2017), provided resistance is not more costly than mating (Gerber & Kokko, 2016). By contrast, obligately sexual females should only resist mating beyond a certain optimum number of copulations (i.e. those required to fertilise all their eggs or obtain sufficiently diverse sperm), or they should vary their level of resistance based on the number of past mates or the quality of their mates (Burke & Bonduriansky, 2017; Arnqvist & Nilsson, 2000) as well as the costs of resistance (Rowe *et al*., 1994). However, the frequency of interactions with males may be an important factor, as it can influence the strength of selection on sexually antagonistic traits as well as their potential costs, and it may thus obscure other patterns (e.g. if obligately sexual females are exposed to males more frequently than facultative females).

We can also expect to find differences in the presence or level of sexually antagonistic traits in mixed‐sex *versus* all‐female populations of facultative parthenogens, but the direction of this difference is less clear. We may find higher levels of female resistance in all‐female populations if effective female resistance is the reason for their lack of males (i.e. if female resistance led to the local extinction of males in these populations, as some models suggest is possible; Burke & Bonduriansky, 2018b, 2019b; Kawatsu, 2013a). Alternatively, if all‐female populations have been devoid of males for many generations while sexually antagonistic coevolution has continued to select for resistance in the mixed‐sex populations, then females in the all‐female populations may be *less* resistant than females from mixed‐sex populations (Kawatsu, 2013b). Thus, establishing the evolutionary history (e.g. time of divergence and historical presence of males) of the populations under study could be an important factor in interpreting findings about the presence and magnitude of sexually antagonistic traits. Unfortunately, we know of no relevant systems where both sexual conflict and recent evolutionary history are well understood.

We review the existing evidence from some of the better‐studied arthropod and vertebrate systems to evaluate predictions of the sexual conflict hypothesis, as well as highlighting promising systems and relevant outstanding questions. We start with the most well‐studied facultative parthenogens (stick insects, Opiliones, and mayflies). Then we review relevant studies in a few other systems where males interact with females capable of parthenogenesis. We consider taxa where tychoparthenogenetic species are common (fruit flies and cockroaches) and highlight a few other promising systems (brine shrimp, ostracods, and lizards). We hope our review will spark increased empirical research on the ecology, life history, and behaviour of these and other parthenogenetic animals.

## II. EVIDENCE AND PROMISING STUDY SYSTEMS

### (1) Facultative parthenogens

#### (a) Stick insects

Parthenogenesis is especially prevalent in Phasmatodea (stick and leaf insects), where it has evolved multiple times (Bullini, 1994; Schwander & Crespi, 2009). This order is particularly promising as a model to study the contribution of sexual conflict to the maintenance of sex because it contains several groups of closely related sexual, asexual, and facultative species, for example in the genus *Timema* (Schwander & Crespi, 2009; Larose *et al*., 2023). Recent work has found evidence of sexual conflict in some facultative species (Burke, Crean & Bonduriansky, 2015; Wilner *et al*., 2025), but the behaviour and life history of many remains unknown. We here focus on five phasmid systems where relevant information has been discovered: the American genus *Timema*, the old‐world genus *Bacillus*, and three facultative parthenogens from Oceania: *Clitarchus hookeri*, *Extatosoma tiaratum*, and *Megacrania batesii*.

Parthenogenesis and sexual behaviour have been studied most in *Timema* stick insects. This genus contains 17 sexual species, four putatively asexual species, and one facultative parthenogen (*T. douglasi*; Larose *et al*., 2023). It also includes several sexual–asexual sister‐species pairs (Schwander *et al*., 2013). However, the lines between obligate sex and parthenogenesis are more blurred than previously thought. Females in several of the sexual species are capable of tychoparthenogenesis to varying degrees when unmated (Larose *et al*., 2023; Schwander *et al*., 2010). Some even produce a few eggs parthenogenetically after mating, and some of these ‘sexual’ species contain very female‐biased populations (Schwander *et al*., 2010). On the other hand, the single confirmed facultative species was believed to be obligately asexual until very recently, when it was shown that males are common in some populations, and that females in some lineages can reproduce both sexually and asexually with high hatching success (Larose *et al*., 2023). Population‐genetic data also suggest that cryptic sex occurs in another of the putatively asexual species (Freitas *et al*., 2023). Moreover, rare, spontaneous males have been found in all but one of the putatively asexual *Timema* species (Schwander *et al*., 2013); these males probably arise through disjunction errors, due to an XX/XO sex‐determination system (Schwander & Crespi, 2009; Schwander *et al*., 2013). Laboratory studies show these rare males are often fertile, and while no copulations with conspecific females have been achieved in the laboratory (Schwander *et al*., 2013), population genetics suggest that these males may breed with conspecific females in some populations (Freitas *et al*., 2023).

*Timema* mating and courtship behaviour are suggestive of sexual conflict, as is their genital morphology. Males possess not just genital claspers, but also an intradextral process that they use to pull the female's subgenital plate open for intromission, and phylogenetic analyses suggest these genitalic traits are subject to continuous selection, consistent with sexual selection or sexual conflict (Arbuthnott *et al*., 2010). While females are capable of rejecting male mating attempts, those of sexual species rarely do so (Arbuthnott & Crespi, 2009), and they often mate multiply in the laboratory (Arbuthnott, Crespi & Schwander, 2015). This multiple mating seems to provide indirect but not direct benefits to females (Arbuthnott *et al*., 2015), and it may be costly due to prolonged mating times (3–5 h) and post‐copulatory mate guarding (several days), during which the male rides on the female's back (Arbuthnott & Crespi, 2009). Furthermore, some *Timema* male courtship behaviours may be coercive. As part of the courtship sequence, before copulation, males rapidly wave their antennae and legs while mounted on the female's back (Arbuthnott & Crespi, 2009). Copulation is always preceded by these ‘courtship’ movements, but they appear to have little bearing on female mate choice (Arbuthnott & Crespi, 2009). Arbuthnott & Crespi (2009) have speculated that these movements may in fact be a coercive behaviour, hindering female crypsis and thus imposing a cost on resisting and delaying copulation.

Females from asexual species of *Timema* have been observed actively resisting mating attempts, and they are much less likely to copulate with males in the laboratory than their sexual counterparts (Schwander *et al*., 2013). It is not entirely clear whether this decrease in copulation is driven by female reluctance to mate, female choosiness, or male choice, as it is accompanied by a reduction in copulatory ‘courtship’. However, further data suggest that female sexual traits have been selected against in these asexual species (Schwander *et al*., 2013). These females tend to exhibit deformed spermathecae (sperm‐storage organs), less‐attractive volatile pheromones, and different and more variable cuticular hydrocarbon (CHC) profiles than their sexual counterparts, and the speed at which these changes have occurred suggests that they are the product of selection rather than neutral processes (Schwander *et al*., 2013). Together, these findings lend some support to the idea that these females may be under selection to avoid or resist mating or fertilisation.

Moreover, experimental crosses suggest that mating with males causes reduced egg viability in facultatively parthenogenetic *Timema douglasi* females from certain lineages, although mating status was confounded with female age in this experiment (Larose *et al*., 2023). Mating seemed to affect hatching success differently for females from different genotypes (which also varied in their propensity to have eggs fertilised), so the authors suggested there may be a trade‐off between the abilities for parthenogenetic and sexual reproduction, such that females that have evolved greater parthenogenetic ability are less effective in reproducing sexually (Larose *et al*., 2023). Such a trade‐off could make mating and fertilisation costly for females that have evolved to reproduce parthenogenetically. There is in fact evidence that barriers to fertilisation have evolved not only in the *T. douglasi* lineage that had higher parthenogenetic ability (Larose *et al*., 2023), but also in other distantly related facultatively parthenogenetic stick insect species: *Bacillus rossius* (Bedford, 1978), *Clitarchus hookeri* (Morgan‐Richards, Trewick & Stringer, 2010; Morgan‐Richards *et al*., 2019; Morgan‐Richards, 2023) and *Megacrania batesii* (Wilner *et al*., 2025).

*Bacillus* is another promising stick‐insect genus. While genetics, cytology, and development are well studied in this group, their behavioural ecology is not, and what is known of their reproductive biology is intriguing. Species in this group have a variety of reproductive modes, including facultative parthenogenesis in *B. rossius*, obligate sex in *B. grandii*, seemingly obligate parthenogenesis in *B. whitei*, *B. atticus*, and *B. lynceorum*, and even hybridogenesis (where fertilisation occurs, but the resulting offspring only pass on their mother's genes when they reproduce) and androgenesis (where offspring only inherit nuclear genes from their father, not their mother) in various hybrid lines [reviewed in Scali (2009) – for reviews of hybridogenesis and androgenesis across taxa see Lehtonen *et al*. (2013) and Schwander & Oldroyd (2016)]. The facultative parthenogen *B. rossius* occurs in both mixed‐sex and all‐female populations, and while mating often improves fecundity and hatching success, many females show barriers to fertilisation (Bedford, 1978). Some females from mixed‐sex populations produce only daughters after mating, and when females from all‐female populations mate with males from mixed‐sex populations, only one in ten offspring is male (Bedford, 1978).

Hybridisation is common in *Bacillus* and has varied consequences (Scali, 2009). Females from the putatively asexual species *B. atticus* can produce some sons when crossed with heterospecific males in the laboratory, but hatching success seems reduced, and while these sons are fertile if the paternal species is *B. grandii*, they are infertile if it is *B. rossius* (Marescalchi & Scali, 2003). By contrast, genetic work on wild populations shows that *B. rossius* females have hybridised with *B. grandii* males and engendered several of the other *Bacillus* species in this way (reviewed in Mantovani, Passamonti & Scali, 1999; Scali, 2009). Some of these hybrids are hybridogenic females (see Table 1): they carry genes from both of their parents, but only pass on their mother's genes in their own gametes, excluding their father's genes from the germline. Each time one of these hybrids backcrosses with a male from the parental species *B. grandii*, that male's genes are incorporated into the offspring soma but are not passed on to grand‐offspring (Mantovani & Scali, 1992). In this case, it is male fitness that is negatively impacted, rather than that of the parthenogens involved, but the situation is more complex. Sometimes, androgenesis occurs (see Table 1) instead of hybridogenesis, and the maternal nuclear genome is the one discarded, essentially producing *B. grandii* offspring but with *B. rossius* mitochondria (Mantovani & Scali, 1992) – for reviews see Mantovani *et al*. (1999), McKone & Halpern (2003) and Schwander & Oldroyd (2016). When androgenesis occurs, the female seems to have no fitness return for her investment in the egg (although her mitochondria do); males thus behave as sexual parasites (Lehtonen *et al*., 2013). However, it is not clear how often androgenesis occurs and results in viable offspring in the wild. Wild individuals with signs of androgenesis have been found (Mantovani, Passamonti & Scali, 2001), but in laboratory crosses, androgenetically produced offspring appear to be rare and have poor survival (Mantovani & Scali, 1992). Sex in *Bacillus* populations thus seems rife with conflict, but mating can have varied and complicated outcomes, and there have been no experiments explicitly testing the fitness impacts of sexual interactions between parthenogens and males.

*Clitarchus hookeri* is an increasingly well‐studied facultative parthenogen from New Zealand, with all‐female populations established accidentally in the UK (Morgan‐Richards *et al*., 2010). Often described as a geographic parthenogen, it forms both all‐female populations and mixed‐sex populations in its native range, some female‐biased and some with approximately equal sex ratios (Morgan‐Richards *et al*., 2010). Crosses between females from all‐female populations and males from mixed‐sex populations have revealed barriers to fertilisation after mating (Morgan‐Richards *et al*., 2010, 2019), which are not found between individuals from different mixed‐sex populations (Morgan‐Richards, 2023). But the mechanisms behind these barriers remain unknown (Morgan‐Richards *et al*., 2010). Interestingly, two populations appear to have recently transitioned from asexuality to sex, and while one of these transitions appears to be the product of invasion by incoming males, the other seems to have stemmed from the spontaneous asexual production of males (Morgan‐Richards *et al*., 2019).

While the fitness impacts of males on *Clitarchus hookeri* females have not been directly investigated, mating behaviour and morphology suggest some conflict is likely. As in many other phasmids, mating occurs after a male mounts a female and, resting on the female's back, uses toothed genital claspers to grasp the female's opercular organ (Myers, Buckley & Holwell, 2015). Females in mixed‐sex populations are often found mating with males (Morgan‐Richards *et al*., 2010) and are sometimes subject to lengthy mate guarding (typically for several hours after mating, often several days) as part of a scramble‐competition mating system (Myers *et al*., 2015). Males guard females before and after mating: resting on or next to the female, grasping her with the legs and/or genital claspers, and sometimes mating again during guarding (Myers *et al*., 2015). *C. hookeri* females can prevent mating passively, by keeping their genital plates closed throughout a male's attempt, or actively, by running and shaking (Myers, Buckley & Holwell, 2016). However, the context in which active resistance behaviour has been observed is more consistent with mate choice or mate recognition than general resistance to mating. Resistance behaviours have been observed in the laboratory (in females from mixed‐sex populations) in response to males whose claspers were experimentally damaged, while females paired with undamaged males mated readily with them (Myers *et al*., 2016). Resistance behaviours have not been observed in wild mixed‐sex populations (Myers *et al*., 2015). In the wild, *C. hookeri* also hybridises extensively with the closely related and recently described *C. tepaki*, whose males have enlarged genital claspers (Myers, Holwell & Buckley, 2017), but the fitness consequences of this hybridisation are unknown (but see Langton‐Myers, Holwell & Buckley, 2019).

There is more evidence of sexual conflict in the facultatively parthenogenetic stick insect *Extatosoma tiaratum*, but the costs of sex in this species also remain unclear due to their context dependence and our lack of knowledge regarding selective pressures in the wild. Interactions with males during development can negatively impact female fitness and hinder the ability to reproduce parthenogenetically: females reared with males as juveniles go on to experience greatly reduced hatching success and reproductive output when laying parthenogenetic eggs (Burke & Bonduriansky, 2019a; Schneider & Elgar, 2010). Furthermore, while sexual reproduction itself is not costly in the laboratory (in longevity, fecundity, hatching success, or nymph survival; Burke *et al*., 2015) and may even improve fecundity (Schneider & Elgar, 2010), switching from parthenogenesis to sex is costly. Females lay fewer eggs and die earlier if they mate after having started to lay parthenogenetic eggs, compared to those who only reproduce asexually (Burke *et al*., 2015; Schneider & Elgar, 2010). However, sexually produced offspring from these females seem to have higher survival than parthenogenetically produced offspring (Burke *et al*., 2015), so it is unclear which reproductive strategy would be more competitive in the wild. Also, impaternate (parthenogenetically produced) females are more prone to wing deformities and are smaller than their paternate (sexually produced) counterparts, but female wings are vestigial in this species, and there is no difference in fluctuating asymmetry between paternate and impaternate females' legs (Burke & Bonduriansky, 2022). Further, impaternate females do not benefit from mating in terms of hatching success, while their sexually produced counterparts do (Burke & Bonduriansky, 2022). But two consecutive generations of asexual reproduction may lead to a reduced immune response (Alavi, Elgar & Jones, 2017). Overall, there is considerable evidence that the costs and benefits of sex are context dependent in *E. tiaratum*. It is unclear whether females would be under selection to avoid sex in wild populations.

Additional work on the sexual behaviour of *Extatosoma tiaratum* in the laboratory is strongly suggestive of sexual conflict over mating. While males are attracted to the scent of females, pre‐reproductive females are repelled by the scent of males, and when disturbed, they secret an ‘antiaphrodisiac’ that repels males (Burke *et al*., 2015). Moreover, in line with the documented cost of switching in this species, once unmated females start laying eggs, their scent seems to change so that males are no longer attracted to them, suggesting an adaptation to avoid switching from parthenogenesis to sex (Burke *et al*., 2015). When males attempt to mate, females exhibit agonistic behaviours, kicking and curling their abdomens, which makes it difficult for males to clasp them (Burke *et al*., 2015). Paternate and impaternate females are equally likely to perform these behaviours, but impaternate females are less likely to receive mating attempts and mate than their paternate counterparts, suggesting that they may be less attractive (perhaps *via* modified pheromones) or more successful in resisting male attempts (Burke & Bonduriansky, 2022).

While *Extatosoma tiaratum* presents tantalising evidence of sexual conflict, the effects of males and sex on females in the laboratory are clearly context dependent, making knowledge of wild populations essential to draw conclusions about the evolution of reproductive modes. Unfortunately, it is very difficult to study *E. tiaratum* in their natural rainforest canopy habitat. Work on another Australian facultative parthenogen, *Sipyloidea larryi* found no costs of mating or switching, but virgin females sometimes struggle to dislodge males (Burke & Bonduriansky, 2018a). Unfortunately, little is known of *S. larryi* wild populations either, making it difficult to interpret these findings.

**Fig. 2:**
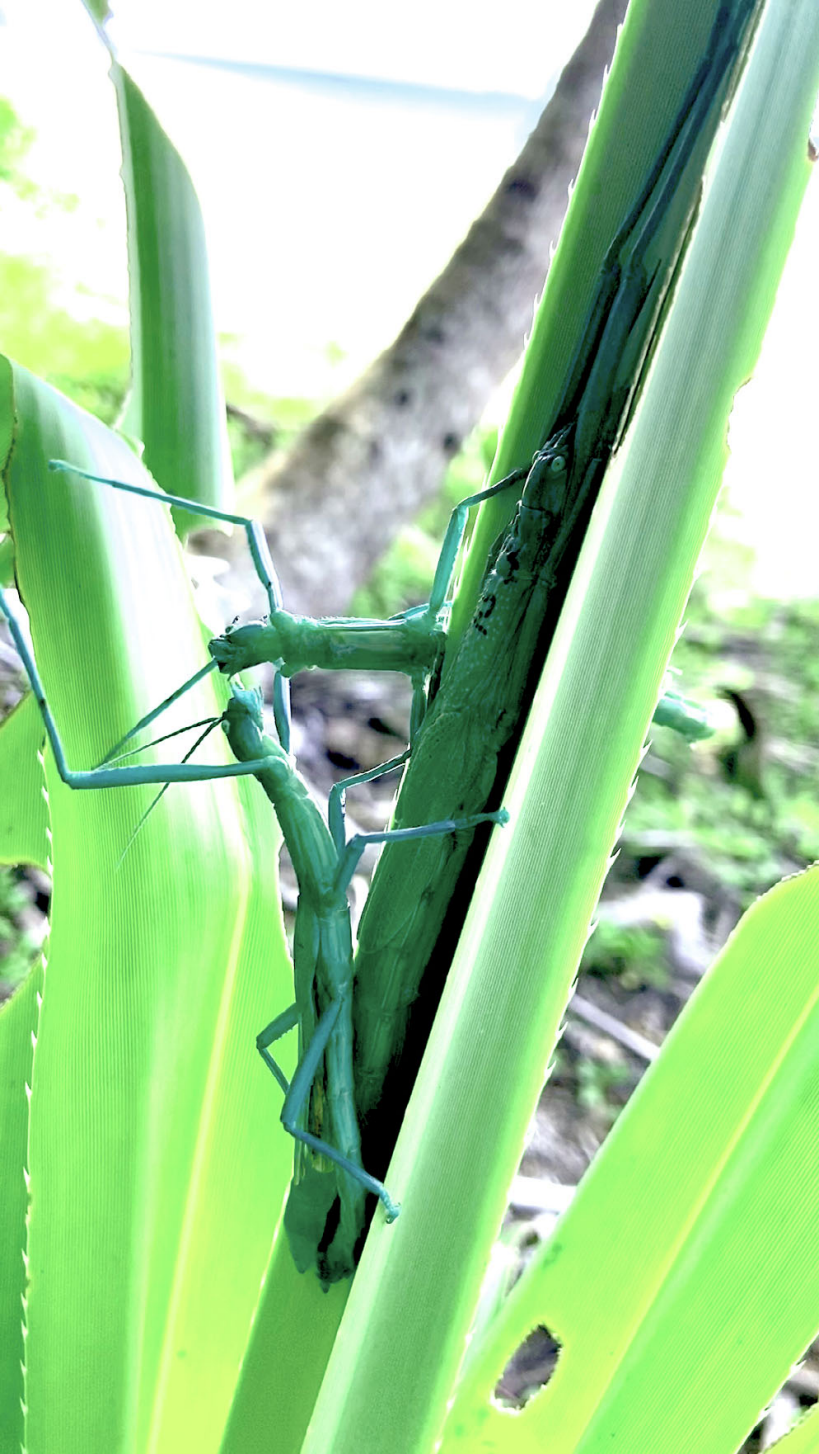
Two males fighting on top of a female peppermint stick (*Megacrania batesii*), a facultative parthenogen from Queensland, Australia. Prolonged mate guarding in this species, and male–male interactions such as this fight, could increase predation risk for females.

There is another Australian stick insect that offers promising opportunities for research on natural populations. *Megacrania batesii* is a facultative parthenogen that forms a mosaic of all‐female and mixed‐sex populations in its natural habitat. The distribution of mixed‐sex and all‐female populations is not explained by ecological factors (Miller *et al*., 2024b), and has remained largely stable over several years of observations, despite the lack of obvious barriers to dispersal between several mixed‐sex and all‐female populations (Miller *et al*., 2024a). The mixed‐sex populations have approximately even sex ratios and feature prolonged mate‐guarding (Boldbaatar *et al*., 2024), where the male rests on the female's back, or next to her, for several days or weeks. Males possess toothed genital claspers capable of grasping females with considerable strength, and they can engage in vicious fights over females, sometimes on top of the females themselves (D. Wilner, personal observations; Fig. 2). There is also some evidence that male guarding behaviour can reduce female foraging rates in certain contexts (Boldbaatar, 2022).

*M. batesii* females from long‐established all‐female populations are resistant to mating and fertilisation. Compared to females descended from mixed‐sex populations, those descended from all‐female populations are less likely to mate with males in the laboratory, and even after mating, they produce most of their offspring from unfertilised eggs (Wilner *et al*., 2025; Wilner, 2025). Findings also suggest that females from all‐female populations have modified chemical signals that could make them less attractive or less detectable to males (Ying *et al*., 2024; Wilner, 2025). This evolved resistance thus involves both pre‐ and post‐copulatory mechanisms, and work with experimental populations in semi‐natural enclosures suggests that female resistance could reduce the probability of male invasion of all‐female populations (Wilner, 2025). There is also evidence of a maternal effect that confers some resistance to impaternate females in mixed‐sex populations (Wilner *et al*., 2025), and might make them more likely to flee from males (Wilner, 2025), similar to the effect found in *E. tiaratum* (Burke & Bonduriansky, 2022). These maternal effects could help females balance the costs of resistance and mating, which are expected to vary based on male‐encounter rates (Gerber & Kokko, 2016). Since paternate females are produced by mated mothers, and impaternate females are typically produced by unmated mothers, paternate females may be more likely than impaternate females to encounter males. A maternal effect causing increased resistance in impaternate females could therefore function to modulate levels of resistance based on anticipated male‐encounter rates and consequent costs.

However, the adaptive significance of female resistance and avoidance is unclear in *M. batesii*. Laboratory studies have not found clear direct costs of mating, and females can benefit from it (*via* increased fecundity and egg viability; Vasconcelos, Adler & Bonduriansky, 2025; Wilner *et al*., 2025). However, these effects of mating on female offspring production appear to be context dependent: they are variable and appear to depend on female genotype and developmental origin (Wilner, 2025; Wilner *et al*., 2025). Additionally, females appear to provision their eggs with more defensive compounds when they reproduce asexually (unmated) than when they reproduce sexually (Vasconcelos *et al*., 2025). This difference in provisioning could indicate that females experience costs from mating or from interacting with males that limit their ability to provision eggs, and it could lead to increased survival of parthenogenetically produced offspring relative to sexually produced offspring. As in other species, it is possible that mating incurs subtle costs, or costs that are only apparent in the wild. The behaviour of these insects suggests that males might impose substantial direct costs on females in the wild (e.g. associated with elevated risk of injury, predation, or parasitism), which could result in net fitness costs for females, especially for resistant females that do not attain counterbalancing reproductive benefits from sex.

Overall, the available evidence strongly suggests that sexual conflict occurs in some phasmids, at least in some contexts. Male behaviour and morphology suggest conflict over mating, and several parthenogenetic phasmids exhibit evidence of female avoidance or resistance to mating as well as barriers to fertilisation. However, the effects of males and mating on female performance and fitness remain understudied, and current evidence suggests context dependence, as well as variation among species and populations. The existence of both all‐female and mixed‐sex populations in many facultative species suggests a role for sexual conflict in tipping the balance between sex and asexuality, and context dependence may help explain why the outcome varies among populations. But much remains unknown about many of these species. To contextualise and interpret current findings confidently, it is essential to understand better whether females are truly able to reproduce successfully both sexually and parthenogenetically in these species, as well as their tendencies to do each in the wild. More work measuring fitness consequences of male–female interactions is also needed, especially in wild populations.

#### (b) Opiliones

The Opiliones, also known as harvesters or harvestmen, are a group of arachnids that constitute another promising taxon for investigation. Many of them exhibit fascinating sexual and parental care behaviours, and there appear to be at least two facultatively parthenogenetic species. The order has gained increasing attention in the last 20 years, but parthenogenesis in Opiliones remains understudied. A recent review of opilionid reproductive biology (Machado & Burns, 2024) lists 18 putatively parthenogenetic species, and indicates that 13 of these are obligate parthenogens, based on the lack or rarity of males (and in a few cases, the additional lack of sperm storage organs in females). However, the reproductive biology and behaviour of these putatively obligate parthenogens remains mostly unknown. The remaining five species are listed as exhibiting geographic parthenogenesis because sex ratio varies across populations or geographical areas, and some locations contain only females. The two best studied of these (*Leiobunum globosum* and *Leiobunum manubriatum*) appear to be facultatively parthenogenetic (Tsurusaki, 1986; Burns, Hedin & Tsurusaki, 2018; Brown, Tsurusaki & Burns, 2021), but little is known about the other three species.

Genital morphology suggests that there are varying levels of sexual conflict over mating in Opiliones (Burns, Hedin & Shultz, 2013; Burns & Shultz, 2015). Unlike other arachnids, male Opiliones (except in Cyphophthalmi) have a specialised intromittent organ (penis), and use their pedipalps for grasping females rather than for sperm transfer (Machado & Macías‐Ordóñez, 2007). Many species also have accessory glands that produce a nuptial gift, and some also have specialised sacs on the end of the penis to store and provide additional nuptial gift secretions (Burns *et al*., 2013; Burns, Hedin & Shultz, 2012). Phylogenetic analyses of leiobunines (i.e. species in *Leiobunum* and closely related genera) in eastern North America suggest that male and female morphology coevolved, and that there have been multiple transitions from more courtship‐based male strategies to more coercive strategies (Burns *et al*., 2013; Burns & Shultz, 2015). These studies found that, in the ancestral state, females had a simple genital opening, and males had penile gift sacs and relatively simple pedipalps. The derived states seem more adapted for conflict: female genital openings have sclerotised barriers, and male penises are sac‐less (and often longer and potentially able to exert more force), and in some of these species, males also have enlarged pedipalps that might be better for clasping females (Burns *et al*., 2013; Burns & Shultz, 2015). Sexual behaviours also appear to differ between the two morphological syndromes (Brown, Marinko & Burns, 2025), but fitness data will be necessary to determine whether these differences correspond to increased levels of conflict.

The genus *Leiobunum* is not monophyletic, and the putative facultatively parthenogenetic Opiliones (*Leiobunum globosum* and *Leiobunum manubriatum*) belong to a Japanese clade that is distinct from the North American leiobunine clade discussed above (Burns *et al*., 2012; Hedin *et al*., 2012), but similar morphological syndromes have evolved in the North‐American and Japanese clades (Hedin *et al*., 2012; Burns & Tsurusaki, 2016). If transitions towards antagonistic morphology have occurred in *L. globosum* and *L. manubriatum*, this could suggest a role for sexual conflict in the evolution of facultative parthenogenesis. However, to interpret morphological variation effectively and confirm that sexual conflict occurs, behavioural data on interactions between males and females is necessary, as well as life‐history data indicating whether these interactions impose costs or benefits for females.

There has been little work regarding sexual conflict in *Leiobunum globosum* and *Leiobunum manubriatum*, and the evidence so far is mixed. Males in these species do lack penial sacs (Hedin *et al*., 2012; N. Tsurusaki, personal communication) and have larger pedipalps than their close relatives, and pedipalps are larger in populations with more female‐biased sex ratios (Burns & Tsurusaki, 2016). If sexually antagonistic selection favours larger pedipalps, these findings would suggest that there is increased sexual conflict in these facultatively asexual species (Tsurusaki, 2004), consistent with the sexual conflict hypothesis. However, females of these species do not have hardened genital barriers like the female leiobunines from North America (Burns & Tsurusaki, 2016), and little is known about these species' reproductive behaviours or the fitness consequences of behavioural interactions between the sexes. Enlarged pedipalps could be the product of sexually antagonistic selection, as their association with transitions to more coercive strategies in North American leiobunines suggests (Burns *et al*., 2013). But large pedipalps could also be selected for by male–male competition if they are used in male–male contests, or by female mate choice if they function as ornaments. Moreover, it may be that shorter, not longer, pedipalps increase male reproductive success in the face of sexual conflict. In the obligately sexual *Leiobunum vittatum* from North America, males with shorter pedipalps are more successful in grasping females, possibly because shorter pedipalps confer a mechanical advantage (Fowler‐Finn, Triana & Miller, 2014). Thus, behavioural and fitness data will be necessary to interpret the morphological findings.

Interestingly, a recent study found that *Leiobunum manubriatum* females produce many offspring parthenogenetically, even when collected as adults from populations with relatively high proportions of males, suggesting that these females may avoid mating or fertilisation (Brown *et al*., 2021). The same study investigated the effect of males on the fitness of *L. manubriatum* females by comparing the fecundity of females from populations with different sex ratios, but did not find evidence of an effect. The authors hypothesised that female avoidance behaviours may mitigate the costs of male harassment in these populations (Brown *et al*., 2021). Avoiding males could also be beneficial because males are brightly coloured, so mating could potentially compromise female crypsis (M. Burns, personal communication). This was an observational study that only measured one fitness proxy, but it seems to support the idea that female avoidance behaviours can allow parthenogenesis to persist in facultative systems by facilitating escape from male harassment. However, to design and interpret experiments testing the sexual conflict hypothesis in these animals, it will be necessary to verify that *L. globosum* and *L. manubriatum* are indeed two facultatively parthenogenetic species, and not a different kind of system (e.g. a complex of obligately sexual and obligately asexual species).

Despite being the two best studied cases of parthenogenesis in Opiliones (Machado & Burns, 2024), there is still little known about *L. manubriatum* and *L. globosum*. Each species consists of mixed‐sex and all‐female populations, and a rearing experiment confirmed that unmated females from both species can reproduce parthenogenetically. But females were sampled from only two locations, and in the case of *L. manubriatum*, both of the sampled populations were all‐female (Tsurusaki, 1986). Moreover, the experiment only tested tetraploid females, but many *L. manubriatum* females in the Japanese Alps (the region with more males) are diploid (Burns *et al*., 2018; Tsurusaki, 1986). Tsurusaki (1986) concluded that both species are likely thelytokous facultative parthenogens, but pointed out that it is also possible that each species is made up of two types of females that reproduce differently (some *via* obligate sex, others *via* obligate asexuality). Nonetheless, recent population‐genetic data seem inconsistent with obligate lineages (Burns *et al*., 2018), and a recent observational study suggests that *L. manubriatum* females from across the range are capable of both sexual and asexual reproduction: DNA sequencing of wild‐collected females and their egg clutches from several locations with different sex ratios uncovered some mixed clutches, where some offspring had sires while their siblings appeared to be parthenogenetically produced (Brown *et al*., 2021). Genomic data thus support the conclusion that these species are facultatively parthenogenetic, but experimental evidence across the range, and in both tetraploid and diploid females, would be ideal.

To complicate matters further, recent molecular work also suggests that *L. manubriatum* is not monophyletic. Some clades appear to be more closely related to other species (*L. globosum* and *L. tohokuense*) than their conspecifics (Burns *et al*., 2018). It seems we may not be looking at just two species in this system. Some authors have even suggested the possibility of different reproductive mechanisms (e.g. gynogenesis) occurring within *L. manubriatum* as well (Burns *et al*., 2018). Molecular data also suggest that some *L. manubriatum* females could be hybridising with *L. globosum* males, and indeed, one such mating event has been observed in the wild (Burns *et al*., 2018). The frequency and fitness outcomes of such hybridisation events remain unknown, and there are no published studies on the sexual behaviour (antagonistic or otherwise) in or between these species, although work in the area is ongoing (N. Tsurusaki, personal communication). Many questions remain, and there are exciting possibilities to explore the effects of sexual conflict. For example, males are rarer in *L. globosum* than *L. manubriatum* (Tsurusaki, 1986). It would be interesting to test whether this difference in sex ratio can be explained by increased female resistance to mating or fertilisation in *L. globosum*. Such resistance could also explain the apparent tendency for *L. globosum* males to mate with *L. manubriatum* females instead. It would also be interesting to track sex ratios of both species over time to see if they are stable or if sex is in the process of being lost.

One leiobunine in which both behaviour and morphology have been well studied is *Leiobunum vittatum*. While it is an obligately sexual species from North America, it may be useful to make predictions about facultative species based on what is known of *L. vittatum*. This species presents the set of seemingly sexually antagonistic traits that have coevolved multiple times in *Leiobunum*: a female pregenital barrier, a lack of specialised nuptial gift sacs on the end of the penis, and enlarged male pedipalps (Burns *et al*., 2013). Males do possess the necessary glands to produce a nuptial gift, and they seem to provide some to females during mating (Fowler‐Finn *et al*., 2014), but the chemistry of the secretion is different from that of species with penial sacs (Kahn *et al*., 2018). This species often exhibits pre‐copulatory wrestling as males attempt to grasp females and copulate with them. But even when securely grasped, females seem to have control over whether intromission occurs or not, and they sometimes reject males by pressing their bodies against the substrate, blocking access to their genital opening (Macías‐Ordóñez, 1997; Fowler‐Finn *et al*., 2014). These male–female struggles could be the result of male coercive attempts, but some authors have argued they may simply be an instance of tactile courtship or assessment. These animals are nearly blind and do not seem to use volatile pheromones, so assessment may only be possible through physical contact (Machado & Macías‐Ordóñez, 2007). *L. vittatum* females also exhibit a set of behaviours consistent with accepting or even courting the male: tapping on the male's pedipalps, grasping his chelicerae and penis, and even appearing to guide the penis toward the genital opening (Machado & Macías‐Ordóñez, 2007; Fowler‐Finn *et al*., 2014; Edgar, 1971).

Studies in wild sexual opilionids suggest highly context‐dependent mating behaviour (Machado & Macías‐Ordóñez, 2007) that is likely to impact females negatively. It would be worth exploring whether parthenogenetic females engage in similar behavioural interactions with males. Again, *L. vittatum* provides a useful example. In part of their range, this species exhibits resource defence polygyny: males fight each other, defend oviposition sites, and guard females after mating, holding the female by a leg and following her around as she peruses the male's site and evaluates various spots for oviposition (Macías‐Ordóñez, 1997). If males and their mating attempts constitute a barrier for females to access oviposition sites, this could result in conflict over mating. But interestingly, the long‐term study that described this mating system also documented many undefended oviposition sites (Macías‐Ordóñez, 1997). It seems puzzling that males would defend a resource that is not limiting, but perhaps these undefended sites are of lesser quality. It would be worth exploring whether *L. globosum* and *L. manubriatum* males also defend oviposition sites. *Leiobunum* females often oviposit alone, but *L. manubriatum* males are frequently found resting on potential oviposition sites, possibly waiting to intercept females (M. Burns, personal communication). In another part of the range of *L. vittatum*, where they coexist with a few other *Leiobunum* species, they do not exhibit resource defence polygyny. Rather, *L. vittatum* in these areas are often found in mixed‐sex clusters, and males do not appear to defend territories or guard females, but interspecific mating attempts and vigorous struggles have been observed (Edgar, 1971). It seems likely that parthenogenetic females in other leiobunine assemblages could also experience harassment from heterospecific males. This would be worth exploring in the context of the sexual conflict hypothesis.

While interactions with males could impose costs on females or limit their ability to reproduce parthenogenetically, there is also the potential for them to provide direct and indirect benefits *via* nuptial gifts or male parental care. Further, since *L. manubriatum* females sometimes continue to produce some unfertilised eggs after mating (Brown *et al*., 2021), some benefits of mating could apply to females' parthenogenetically produced offspring as well as their sexually produced offspring. Nuptial gifts are common and likely ancestral in leiobunines (Burns *et al*., 2013), but to our knowledge, nuptial gifts have not been documented in the facultatively parthenogenetic species (*L. manubriatum* and *L. globosum*). Males in many species of Opiliones also provide extensive (and sometimes exclusive) parental care by guarding eggs from predation, and females in some of these species court males (Machado *et al*., 2015). To our knowledge, paternal care has not been documented or investigated in leiobunine harvesters. *L. manubriatum* and *L. globosum* do not build nests, and no evidence of parental care has been observed in these species (M. Burns, personal communication), but it seems worth exploring whether nuptial gifts or other benefits of mating occur. Given the dearth of behavioural work on *L. manubriatum* and *L. globosum*, many intriguing possibilities remain.

#### (c) Mayflies

Parthenogenesis is extremely widespread in mayflies (Ephemeroptera), and it is present to some degree in more than half of the 136 mayfly species where reproductive mode has been studied (mostly in the Northern Hemisphere; Liegeois, Sartori & Schwander, 2021). There are both putatively obligate and facultatively parthenogenetic species, as well as varying degrees of tychoparthenogenesis (Liegeois *et al*., 2021; Funk, Sweeney & Jackson, 2010; Degrange, 1960). It has been argued that parthenogenesis may be an adaptation to mate limitation and/or incomplete fertilisation in these famously short‐lived insects (Liegeois *et al*., 2021; Funk *et al*., 2010) which are thought to be under intense pressure to lay eggs quickly (Araújo, Dias & Serrão, 2021). Parthenogenesis may allow females to bypass mating and associated costs entirely. In fact, this strategy appears to have reached an extreme in the species *Eurylophella oviruptis*. In this all‐female species, not only are females parthenogenetic, but many have been found to ‘burst’ as soon as they emerge as subimagos (subadults) at the water surface, filling their gut with air until their abdomen ruptures, spilling out their eggs (along with their guts) before ever taking flight (Funk, Jackson & Sweeney, 2008).

Mayflies are a fascinating, widespread, and very promising group for the study of facultative parthenogenesis, but much remains unknown regarding their behavioural ecology. We found no studies on the sexual behaviour of parthenogens, nor studies specifically investigating sexual conflict or sexual selection in the context of parthenogenesis in this order. However, growing interest in mayfly parthenogenesis is generating interesting findings. There have been several screening and rearing experiments verifying females' ability to reproduce sexually and asexually in multiple species (reviewed in Liegeois *et al*., 2021), which have been compiled into a useful database (Liegeois, Sartori & Schwander, 2020). Mating tends to improve hatching success in facultatively parthenogenetic and putatively facultative mayflies as well as in tychoparthenogenetic species, but the ability of putatively obligate parthenogens to reproduce sexually is rarely tested (Liegeois *et al*., 2021).

Interestingly, a recent study in a putatively facultatively parthenogenetic mayfly found that in the wild, population sex ratios are negatively correlated with density, and repeat surveys suggested they were stable over time (Liegeois, Sartori & Schwander, 2023). This pattern could be the result of mate limitation favouring parthenogenesis, or of male harassment hindering parthenogenesis, or both (Liegeois *et al*., 2023). The same study found a correlation between population sex ratios and the ability of females to reproduce asexually in the laboratory (i.e. the hatching success of unfertilised eggs was higher in females from more female‐biased populations). Experiments will be necessary to determine the causal relationships between all these variables, but these findings are consistent with the idea that male harassment limits the ability of females to reproduce parthenogenetically and has thus hindered the evolution of parthenogenesis in high‐density populations. Females are expected to experience increased harassment at higher population densities due to increased male encounter rates. If this density‐dependent harassment limits females' ability to reproduce parthenogenetically, it could keep population sex ratios even, and it may have led to loss of parthenogenetic ability over time (vestigialisation) in those populations. Conversely, at low‐population densities, females may be better able to escape male harassment and reproduce asexually (leading to female‐biased sex ratios), so there may have been more opportunity for selection to improve parthenogenetic ability in those populations, leading to higher hatching success in these females' unfertilised eggs. Of course, experiments and studies of the behaviour and life‐history of these animals will be necessary to eliminate alternative explanations. The patterns are also consistent with the idea that mate limitation selects for parthenogenetic capability. Further work testing unique predictions from the sexual conflict hypothesis would be informative.

### (2) Promising taxa with frequent tychoparthenogenesis

#### (a) Drosophila

Despite *Drosophila* (fruit flies) being one of the most studied genera on the planet (Zuk *et al*., 2014), the prevalence of true facultative parthenogenesis remains poorly known in this group (Markow, 2013; Sperling & Glover, 2023). Of the approximately 50 *Drosophila* species that have been screened so far, most are capable of some level of parthenogenesis, and one is obligately parthenogenetic. However, rather than each female being able to use both sexual and asexual reproduction flexibly, the so‐called ‘facultative’ *Drosophila* species exhibit only tychoparthenogenesis, with vanishingly low frequencies and success rates of parthenogenetic reproduction (Sperling & Glover, 2023). Even so, artificial selection experiments have demonstrated that the capacity for parthenogenesis can evolve quite rapidly in *Drosophila* (Carson, 1967; Stalker, 1954). It is therefore puzzling that so many *Drosophila* species exhibit only extremely low rates of parthenogenesis. Although genetic and/or physiological constraints are likely to play a part (Engelstädter, 2008), research on mating costs and sexual behaviour in these tychoparthenogenetic species could provide additional insights.

Interestingly, the evolution of parthenogenesis appears linked to a decrease in female receptivity in *Drosophila* flies in the laboratory. Through artificial selection and crossing of different lines, Carson (1967) was able to increase drastically the rate of parthenogenesis in *Drosophila mercatorum* and maintain all‐female colonies. Over the generations, females in these colonies became less likely to mate if they were placed with males, even though the researchers had not been selecting for this trait (Carson, Chang & Lyttle, 1982; Carson, Teramoto & Templeton, 1977). Furthermore, the females no longer derived a fecundity benefit from mating compared to bisexual controls, although they did not seem to incur a cost either (Crews, Teramoto Linden & Carson Hampton, 1985). It was not clear whether the reduction in mating stemmed from female reluctance or from the females being unattractive to males (e.g. due to a change in pheromones resulting from relaxed selection on sexual signals). These mechanisms would be worth disentangling because, while the immediate outcome may be the same (reduced mating), resistance and avoidance may have different costs in wild populations. A link between parthenogenetic ability and reduced mating, combined with a lack of benefits of sex, could promote the establishment of all‐female populations in the wild (Burke & Bonduriansky, 2019b). And yet, while parthenogenetic ability varies geographically in *D. mercatorum* (Kramer & Templeton, 2001), parthenogenesis does not appear to be common in any wild populations. Developmental and genetic constraints may explain this (Markow, 2013; Engelstädter, 2008), but the role of male coercion has not been explored.

Sexual conflict over mating and male harm (e.g. through seminal proteins) have been well documented in *Drosophila melanogaster* (Bateman, 1948; Chapman *et al*., 1995), and it is tempting to assume that interactions with males would also have negative fitness effects on facultatively parthenogenetic *Drosophila* females. However, as cautioned by Zuk *et al*. (2014), it is unwise to make such assumptions because sexual selection is known to generate diverse traits and mating systems, even across closely related species, and some aspects of *D. melanogaster* biology appear to be rather atypical and variable (Zuk *et al*., 2014). There is evidence that multiple mating increases female mortality in *D. mercatorum* (Ikeda 1974 cited in Kramer & Templeton 2001), but experiments on congeners *D. simulans* and *D. pseudoobscura* suggest that females in these species may benefit from multiple mating rather than being harmed by it (Gowaty *et al*., 2010; Taylor *et al*., 2008). Additionally, the costs and benefits of multiple mating and seminal proteins are likely to be highly context dependent (Bonduriansky, 2014). Furthermore, animals from laboratory‐bred lineages may exhibit very different sexual behaviours from wild populations, as a consequence of artificial selection and genetic drift, and sexual selection may operate differently in these lineages. For example, some strains of domestic canaries (*Serinus canarius*) produce very different songs from wild canaries and have reduced hearing (Güttinger, 1985; Wright *et al*., 2004). It will therefore be necessary to test whether males are harmful in species of interest, and to disentangle which contexts determine the relative costs and benefits of mating, and also to study natural populations. On the other hand, recent work has produced a genetically engineered strain of *Drosophila melanogaster* that is capable of parthenogenesis (Sperling *et al*., 2023). This exciting development opens the door for new experimental work on parthenogenesis, leveraging the ample knowledge base of sexual selection and sexual conflict in this model organism.

#### (b) Cockroaches

Several species of cockroaches exhibit tychoparthenogenesis (Roth & Willis, 1956; Brown, 1973), but no truly facultative species have been discovered to date. Instead, where parthenogenesis has been discovered, only a subset of the females tested have been able to reproduce parthenogenetically, with varying levels of success. However, stringent screenings for parthenogenetic ability have only been performed for a few species. *Phyllodromica subaptera*, for example, is promising because only some populations contain males; while many others are all‐female (Knebelsberger & Bohn, 2003). Crossing and rearing experiments will be necessary to determine whether the females in this species are facultatively parthenogenetic or whether different populations engage in different modes of reproduction. Furthermore, even in species where parthenogenetic ability is relatively well studied (e.g. *Nauphoeta cinerea*), researchers tend to assume that parthenogenesis only occurs in unmated females, without checking whether the clutches of mated females include some parthenogenetically produced daughters, like the mixed clutches discovered in mayflies, stick insects, and Opiliones (Brown *et al*., 2021; Schwander *et al*., 2010; Ball, 2001).

While true facultative parthenogenesis has not yet been found in cockroaches, there is an interesting clade that includes both obligately parthenogenetic and obligately sexual lineages. Roaches in the *Pycnoscelus surinamensis/indicus* complex (formerly all known as *Pycnoscelus surinamensis*) occur in both mixed‐sex and all‐female populations across the tropics (Roth & Willis, 1961; Parker *et al*., 1977), but there is currently no evidence of facultative parthenogenesis in these roaches. Males from mixed‐sex populations do not appear to fertilise any eggs when housed with females from all‐female populations (i.e. these females produce no sons, or extremely rare sons, even when sperm is transferred), suggesting that those females are obligately asexual (Roth & Willis, 1961; Roth, 1967). Meanwhile, females from sexually reproducing lines appear unable to reproduce parthenogenetically (Roth & Willis, 1961). Given the evidence of reproductive isolation between sexually and parthenogenetically reproducing lineages, Roth (1967) split the taxon into two species: reserving the name *P. surinamensis* for parthenogenetic lineages and naming the sexually reproducing lineages *P. indicus*. However, *P. surinamensis* in its new definition may be polyphyletic: parthenogenetic lineages appear to have arisen multiple times from *P. indicus* colonising different areas (Parker *et al*., 1977). Moreover, the capacity for females to reproduce *via* both sex and parthenogenesis has only been tested in individuals from a few locations.

Interestingly, sexual and parthenogenetic cockroaches in the *Pycnoscelus surinamensis/indicus* complex appear to coexist in Indonesia and Hawaii (Roth & Willis, 1961; Roth, 1967; Roth, 1974), and it is likely that they interact. Some parthenogenetic lineages are triploid, and may have arisen from backcrosses between diploid parthenogenetic and sexual lineages (Parker *et al*., 1977). While successful interbreeding between sexual males and females from parthenogenetic lineages has not been documented, females from asexual populations apparently continue to produce sex pheromones (Barth, 1965), and males attempt to mate with them in the laboratory (Roth & Willis, 1961). However, these females have been observed resisting those mating attempts, and examinations of their spermathecae have revealed that they are less likely to receive sperm than females from sexually reproducing lineages (Roth & Willis, 1961). Moreover, while mating does not appear to result in fertilisation for these females (only daughters are produced), it has been shown to impact their fitness negatively by reducing the number of hatchlings they produce relative to their unmated counterparts (Roth & Willis, 1961). It would be interesting to investigate why mating reduces parthenogenetic output, and how males affect parthenogenetic females in the wild. Where they overlap, females capable of parthenogenesis appear to be less common than sexually reproducing females (Roth & Willis, 1961; Roth, 1967). Perhaps parthenogens are rare in such communities due to the negative impacts of males on their fitness.

Two cockroach species with tychoparthenogenesis have been well studied in the laboratory for decades and are also promising systems to study the evolution of parthenogenesis: *Nauphoeta cinerea* and *Periplaneta americana*. Most aspects of the biology of *P. americana* have been intensely studied due to its cosmopolitan distribution and pest status, but parthenogenesis and sexual selection are better understood in *N. cinerea*. In *N. cinerea*, parthenogenetic ability is very variable (Corley & Moore, 1999), and heritable (Corley, Blankenship & Moore, 2001). Sexual selection has also been well studied in this species; there have been studies on mating behaviour, mate choice, and sexual conflict (e.g. Clark, DeBano & Moore, 1997; Roth, 1964; Moore *et al*., 2001). But while there is evidence of sexual conflict and manipulative males in *N. cinerea* (Moore, Gowaty & Moore, 2003; Montrose, Harris & Moore, 2004), we still do not know how males affect females' ability to reproduce parthenogenetically. Interestingly, females feed on a male secretion during courtship (Roth, 1964), and exposure to male odours may actually increase the reproductive output of females reproducing parthenogenetically (Moore & Moore, 2003); mating could thus provide females with direct benefits. Physical coercion and harassment by males seem unlikely to play an important role in this species because females initiate the mating sequence (by approaching and mounting a male when he releases attractive pheromones), and females appear to control most stages of courtship (Clark *et al*., 1997; Roth, 1964). However, attractive male pheromones could have evolved *via* exploitation of a sensory bias (see Ryan, 1990; Christy, 1995; Arnqvist, 2006). Other male traits (such as dispersiveness) could also contribute to preventing the further evolution of parthenogenesis, especially since the low success rate of parthenogenesis means that females are likely under strong selection to mate, given the chance.

It is interesting that several cockroaches exhibit tychoparthenogenesis, but only *P. surinamensis* is capable of highly effective parthenogenesis. As in *Drosophila*, researchers studying tychoparthenogenetic cockroach species have concluded that developmental constraints have prevented the further evolution of asexuality in this group (Corley & Moore, 1999). Indeed, the presence of such constraints is clear in both *Drosophila* and cockroaches, but it is not clear why these constraints persist in most but not all species in each group. Most parthenogenetic lineages of *P. surinamensis* are in its introduced range, but is this because mate limitation imposed very strong selection for parthenogenetic ability there, or because the absence of males removed a constraint, or both? Does parthenogenetic ability remain poor in the other species and in the *P. indicus* native range because male behaviour (e.g. coerciveness, dispersiveness, or sensory exploitation) limits the opportunity for females to reproduce parthenogenetically, thus limiting the opportunity for selection to act on parthenogenetic ability?

### (3) Other promising systems where parthenogens interact with males

#### (a) Brine shrimp

The genus *Artemia* (brine shrimp) presents another promising system. These small crustaceans are very well studied in some ways (e.g. in the context of aquaculture, and as a model system in toxicology; Nunes *et al*., 2006), and they are well suited for laboratory experiments. They are also widespread, and some parthenogenetic *Artemia* coexist with sexual ones (Browne & MacDonald, 1982; Zheng & Sun, 2013). However, parthenogenetic *Artemia* remain poorly understood. While they are commonly considered obligately asexual, recent work suggests they may not all be (Boyer *et al*., 2021). Alarmingly, while all parthenogenetic *Artemia* have so far been referred to as *Artemia parthenogenetica* in the literature, mounting evidence shows that this name encompasses several different species, from different branches of the *Artemia* phylogeny (Innes & Dufresne, 2020), and various researchers over the years have questioned the validity of this species name (Asem *et al*., 2023). This makes it difficult to interpret and synthesise existing findings on *A. parthenogenetica*, as different studies may be using completely different animals.

While the sexual behaviour of parthenogenetic *Artemia* remains virtually unstudied, ecological evidence suggests these females may be negatively impacted by interactions with sexual lineages. The invasive sexual species *A. franciscana* is displacing native parthenogenetic *Artemia* in Eurasia (Amat *et al*., 2005, 2007; Zheng & Sun, 2013). *A. franciscana* males do not appear to be very choosy: they have been observed attempting to mate with heterospecific females (including from other genera) and even with inanimate objects (Rogers, 2002). It is therefore likely they would attempt to mate with parthenogenetic *Artemia*. There is also some evidence suggestive of sexual conflict in *Artemia franciscana* (Rode, Charmantier & Lenormand, 2011). Males have very enlarged antennae that they use to clasp females, and females apparently struggle when approached and clasped (Rogers, 2002; Tapia *et al*., 2015). Moreover this species seems to exhibit prolonged mate guarding, as males have been seen holding females for several hours after mating (Rogers, 2002). Parthenogenetic *Artemia* may thus be subject to harassment and costs of mate guarding from sexual males where they overlap. *Artemia* males of other species have been found mating with putatively asexual females in the laboratory (Amat, 1983). Furthermore, rates of harassment experienced by parthenogenetic females could be exacerbated by the fact that sexual *Artemia franciscana* plastically adjust their offspring sex ratio based on adult sex ratio (Lievens *et al*., 2016). They produce more sons when they perceive more females (presumably *via* chemical cues), regardless of whether the excess females are conspecifics or heterospecifics from an asexual lineage. This results in a large surplus of males where *A. franciscana* overlap with all‐female populations of parthenogenetic *Artemia* (Lievens *et al*., 2016). The resulting high encounter rate could lead to excessive costs to females from harassment and/or resistance (Gerber & Kokko, 2016), especially if mating confers no benefits, which would be expected for obligately asexual females.

While parthenogenetic *Artemia* females are commonly considered obligately asexual, recent findings suggest that reproductive mode is more flexible than it seems, in at least some of these animals (Boyer *et al*., 2021). Laboratory work has shown that some rare asexually produced *Artemia* males can be fertile and successfully interbreed with female *Artemia* from sexual species (Bowen *et al*., 1978), but not all can (MacDonald & Browne, 1987). Furthermore, these males can pass on the ability to reproduce parthenogenetically to their offspring (Maccari *et al*., 2014), and some of these offspring are capable of both sexual reproduction (backcrossing with the parental sexual species) and asexual reproduction (Boyer *et al*., 2021). In other words, some of these hybrid *Artemia* can be facultatively parthenogenetic. Several sites containing both sexual and putatively asexual *Artemia* have been documented (Amat *et al*., 2007; Zheng & Sun, 2013), and they may become more common as invasive *A. franciscana* continues to spread in the native range of parthenogenetic *Artemia*. Additionally, while asexually produced *Artemia* males are rare, they have been documented in several populations. It is therefore likely that hybrid lineages exist in the wild. Given that hybrid females can be facultatively parthenogenetic, there may be facultatively parthenogenetic *Artemia* in existence, waiting to be screened and studied.

#### (b) Ostracods

Ostracods or seed shrimp are tiny aquatic crustaceans whose bodies are entirely enclosed in a shell. The class Ostracoda is of special interest to researchers of the evolution of sex because it contains a lineage of putatively ancient asexuals (Schön, Rossetti & Martens, 2009). But parthenogenesis has also evolved more recently and repeatedly in ostracods (Innes & Dufresne, 2020), possibly more times than in any other group of animals (Chaplin, Havel & Hebert, 1994). Most interestingly, asexual ostracods are often sympatric with closely related sexual lineages, and there is evidence of gene flow between some sexual and parthenogenetic lineages (Turgeon & Hebert, 1995). In fact, many obligately asexual lineages seem to have arisen through hybridisation between parthenogenetic females and males of closely related species (Chaplin *et al*., 1994).

It is often unclear whether putatively asexual ostracods are obligate or facultative, however. In many cases, a species is considered asexual simply because no males have ever been found; breeding experiments to confirm asexuality are rare. Moreover, putative asexuals are often found in the same bodies of water as closely related sexual species, and genetic data suggest that many of them interbreed with those sexual relatives (Turgeon & Hebert, 1995). Some putative asexuals have turned out not to be unisexual at all. Males have been found in one population of the putative asexual *Cypricercus fuscatus*, and genetic analysis of the females at that location suggested that some of them were reproducing sexually (Turgeon & Hebert, 1995). On the other hand, some ostracods have been considered facultative parthenogens because they are found in both all‐female and mixed‐sex populations. But few of these potentially facultative systems have been investigated further, and each of the best‐studied ones (*Eucypris virens* and *Candonocypris novaezelandiae*) appears to be a complex of cryptic sexual and asexual species, rather than a single species where each female is facultatively parthenogenetic (Chaplin, 1992; Innes & Dufresne, 2020; Bode *et al*., 2010). While several studies have examined the geographic distributions of putatively sexual and asexual ostracods, and several others have examined their population genetics (reviewed in Innes & Dufresne, 2020), very few have directly studied their reproduction or tested their ability to reproduce using each mode (Chaplin, 1992). Even fewer have examined behavioural interactions between males and putative parthenogens.

Nonetheless, evidence does suggest that male ostracods attempt to mate with females capable of parthenogenesis, and sometimes succeed. Genetic evidence suggests that asexually reproducing lineages of *Eucypris virens* occasionally hybridise with their sexual relatives (Schön *et al*., 2000). There have also been instances of *E. virens* males copulating with parthenogenetic females in the laboratory (Horne, Danielopol & Martens, 1998) and of sperm found in the reproductive tracts of parthenogenetic females (Schmit *et al*., 2013). In another ostracod that also exhibits geographic parthenogenesis (*Limnocythere inopinata*), rare males have been produced asexually in the laboratory and have unsuccessfully attempted to copulate with parthenogenetic females (Yin, Geiger & Martens, 1999). Unfortunately, most observations of parthenogenetic reproduction and of sexual interactions between ostracod males and parthenogenetic females are anecdotal, and the incidence and fitness impacts of such interactions remain unknown. However, hybridisation appears to contribute to the high genetic diversity of many putatively asexual ostracods (Innes & Dufresne, 2020; Chaplin *et al*., 1994), and this hybridisation‐induced diversity has been hypothesised to contribute to the proliferation and success of ostracod parthenogens (Chaplin *et al*., 1994; Schön *et al*., 2000).

Ostracods are a promising group to investigate the effect of males on parthenogenesis, and some evidence is suggestive of potential sexual conflict. Ostracod courtship is characterised by males approaching or pursuing females, touching them, and clasping them, and copulation then begins once the female slows or ceases movement (Horne *et al*., 1998). Researchers have interpreted this behaviour as acceptance on the female's part (Horne *et al*., 1998), but it could reflect male coercion rather than female mate choice; further studies are needed to determine this. Moreover, ostracods have notoriously exaggerated male secondary traits (e.g. large paired hemipenes and giant sperm), and they employ diverse and sophisticated clasping mechanisms (Horne *et al*., 1998), which could indicate a history of intense postcopulatory competition and sexual conflict. Some ostracod males also clasp and guard juvenile females (Danielopol, 1977). Considering these trends in behaviour and morphology, and given that sexual and asexual ostracods are often sympatric, interactions between ostracod males and asexual females have the potential to be costly to parthenogenetic females. But males may also be under selection to avoid mating with parthenogenetic females. *E. virens* females from asexually reproducing clades in the species complex were less likely to copulate with males than their sexual counterparts in a laboratory experiment (Schmit *et al*., 2013). It was unclear whether this difference was driven by female or male choice, but the authors argued that mating with asexual females would be costly for *E. virens* males because their large sperm are limited and expensive, generating selection for male discrimination against such females. However, this may not be the case if females are facultatively rather than obligately asexual. Interactions between male and parthenogenetic female ostracods have the potential to have both positive and negative impacts on both parties, and they likely happen frequently in the wild. Testing the sexual conflict hypothesis in ostracods will require further knowledge of these animals' life histories, their behaviour, their true abilities to reproduce sexually and asexually, and their interactions with each other in the wild.

#### (c) Lizards

Among vertebrates, there are no well‐studied facultative parthenogens, but lizards are a promising group to study the sexual conflict hypothesis. There are multiple gynogenetic (or pseudogamous) vertebrate species (see glossary in Table 1), made‐up of females that produce offspring without genetic contribution from a father, but still need to mate to do so, such as Amazon mollies (Hubbs, 1964). And rare events of parthenogenesis have also been documented across sexual species of fish, reptiles, and amphibians, but all‐female species where every female is capable of reproducing in the complete absence of males only occur in lizards and snakes (Neaves & Baumann, 2011; Vrijenhoek *et al*., 1989; Moreira, Fonseca & Rojas, 2021). The term facultative parthenogenesis is often used in the vertebrate literature as a synonym of tychoparthenogenesis (reviewed by Lampert, 2009), but to our knowledge, true facultative parthenogenesis has not been conclusively demonstrated in any vertebrate species. However, a recent study found evidence that is suggestive of facultative parthenogenesis in captive tropical night lizards (*Lepidophyma smithii*): in this study, mated females produced broods that contained some offspring that developed from unfertilised eggs (Kratochvíl *et al*., 2020).

Lizards are also a promising taxon to investigate the effects of sexual interactions on obligate parthenogens. Several parthenogenetically reproducing all‐female species have been studied across eight squamate families, with particularly well‐known examples in the lacertid genus *Darevskia* (rock lizards), in *Aspidoscelis* whiptail lizards (formerly part of *Cnemidophorus*), in *Gymnophthalmus* tegus, in *Leiolepis* butterfly lizards, and in *Hemidactylus* geckos (Maslin, 2015; Tarkhnishvili, Murtskhvaladze & Anderson, 2017). Most of the asexual lizard species have arisen through hybridisation between obligately sexual species, and although many of the asexual species occupy different geographical areas from their sexual relatives, several do overlap (Tarkhnishvili *et al*., 2017). Sympatric zones are particularly well documented in the genus *Darevskia* (Petrosyan *et al*., 2020), and in this genus, the females of parthenogenetic lineages often mate and hybridise with males of sexual species (Carretero *et al*., 2018; Danielyan, Arakelyan & Stepanyan, 2008).

Nonetheless, we found little detailed research on the behavioural interactions between these sexual and asexual lizards, nor much work on the fitness consequences of these interactions for parthenogenetic females. This provides promising opportunities for future work. What we do know so far is tantalising. Genetic and morphological studies suggest that at least some of the resulting hybrids are sterile (Danielyan *et al*., 2008; Freitas *et al*., 2019), which would make mating costly for parthenogenetic females. Furthermore, courtship in lizards often appears to have aggressive components, for example, male lacertids and geckos bite their mates during courtship and mating (In Den Bosch & Zandee, 2001), and exhibit these behaviours towards parthenogens (Dame & Petren, 2006; Galoyan *et al*., 2025). In fact, ‘copulation marks’ (scars from such bites left on females' abdomens) are used as a diagnostic of whether a lacertid female has mated in the past, and such marks have been found in parthenogenetic *Darevskia* females that are sympatric with bisexual species (Carretero *et al*., 2018; Galoyan *et al*., 2025). Male–female pairs of *Darevskia* lizards exhibit both male aggressive and coercive behaviour (chasing, restraining, and biting females) and female resistance behaviour (fleeing, biting males), and males have been observed biting and mating with parthenogenetic females in the wild (although they prefer conspecifics; Galoyan *et al*., 2025); females do not appear to bite each other in this way (Carretero *et al*., 2018). Unless fertilisation yields very large fitness benefits for offspring, it is likely that interactions with males are costly to these parthenogenetic females, and sexual conflict may be intense.

*Hemidactylus* house geckos are another interesting system. Several species are invasive and widespread, including the sexual species *Hemidactylus frenatus* and the parthenogenetic (all‐female) species *H. garnotii* (Weterings & Vetter, 2018). There is evidence that sexual *H. frenatus* displace parthenogenetic *H. garnotii*, as well as another parthenogenetic gecko (*Lepidodactylus lugubris*), where they co‐occur; although *H. garnotii* in their turn displace other sexual species (Weterings & Vetter, 2018; Case, Bolger & Petren, 1994). A laboratory experiment found that males courted these parthenogenetic females as frequently and more intensely than they courted conspecific sexual females, and also copulated with them (Dame & Petren, 2006). However, these behaviours were not consistent across studies (Bolger & Case, 1992), and did not reduce female feeding behaviour (Dame & Petren, 2006). *H. frenatus* males also behave aggressively towards parthenogens, and this aggression has been hypothesised to contribute to the parthenogens' displacement (Bolger & Case, 1992), but it is unclear whether male aggression towards parthenogens is mainly a sexual or territorial behaviour.

Interestingly, there are also documented cases of rare parthenogenetically produced males in asexual lizard species, and some evidence suggesting that such males may be fertile and hybridise with females of sexual species (Petrosyan *et al*., 2020). More research on these rare males and their potential impact on the fitness of parthenogenetic females is needed. There is the potential for males to have a positive impact on the reproduction of parthenogens. For example, pseudocopulatory behaviour between female conspecifics in asexual *Aspidoscelis uniparens* (formerly *Cnemidophorus uniparens*) whiptail lizards can promote ovulation and egg laying, just as copulation with sterile males does in their sexual counterparts *A. inornatus* (Crews, Grassman & Lindzey, 1986). Perhaps behavioural interactions between parthenogenetic females and rare males, or males of closely related sexual species, could have a similar fertility‐boosting effect, so fitness effects of males need not be entirely negative.

## III. DISCUSSION

The evidence reviewed above suggests the possibility of sexual conflict in several facultatively parthenogenetic species, showing that sexual interactions could impose direct costs on facultatively and obligately parthenogenetic females. We summarise the most relevant evidence in Table 2. It is clear that much remains unknown about parthenogenetic animals and their life histories, and very few studies have set out to test the sexual conflict hypothesis. However, some behavioural and morphological traits are suggestive of a history of sexually antagonistic coevolution in relevant taxa (e.g. in Opiliones and stick insects), and there are documented cases of mating and exposure to males imposing direct costs on facultatively parthenogenetic females. It is also clear, however, that the relative costs and benefits of sexual and parthenogenetic reproduction can be complex and context dependent. For example, in *E. tiaratum*, costs and benefits appear to depend on female developmental origin and reproductive status (Burke & Bonduriansky, 2022, 2019a; Burke *et al*., 2015). To understand the prevalence of sexual conflict in these systems, and its consequences for the evolution of parthenogenesis, we will need a better understanding of the life history and behaviour of these animals in the wild, and we will need to assess how different fitness components are impacted in a variety of ecologically relevant contexts.

**Table 2:** Summary of evidence regarding the sexual conflict hypothesis. Each row lists relevant evidence from one taxon (limited to taxa where a substantial proportion of females are known to be capable of parthenogenesis, and there is some evidence relevant to the sexual conflict hypothesis). *Facultative parthenogenesis*: whether true facultative parthenogenesis has been demonstrated in the taxon; if not, what other relevant form of parthenogenesis has been studied. *Relevant genital morphology*: morphology/physiology suggestive of sexual conflict in species with parthenogenesis. *Relevant behaviour*: behaviour suggestive of sexual conflict in facultatively parthenogenetic species or in parthenogens. *Fertilisation barriers*: evidence that facultative or thelytokous parthenogenesis is associated with a reduction in fertilisation success. *Fitness effects within species*: effects of interactions with conspecific males on the fitness of females of facultatively parthenogenetic species, or of females reproducing parthenogenetically, or parthenogenetically produced females (note that by definition, in tychoparthenogenetic species, most females benefit from mating at least once). *Fitness effects on ‘obligate’ parthenogens*: effects of interactions with males from sexual lineages on the fitness of putatively obligate parthenogenetic females. In the fitness effects columns, we indicate the type of interaction studied (e.g. mating), whether there was a positive effect (+, green), negative effect (−, red), no significant effect (n, blue), or context‐dependent effect (cd, orange), and the type of fitness proxy (e.g. fecundity, viability, longevity, etc.). Note that the term impaternity refers to whether the reproducing female was paternate or impaternate (i.e. whether she was produced sexually or asexually). Grey shaded cells indicate a lack of relevant studies in parthenogens.

Taxon	Facultative parthenogenesis?	Relevant genital morphology	Relevant behaviour	Fertilisation barriers	Fitness effects within species	Fitness effects on ‘obligate’ parthenogens
Stick insects	Yes	Claspers and intradextral process under continuous selection across *Timema* (Arbuthnott *et al*., 2010) Deformed/reduced spermathecae in asexual species (Schwander *et al*., 2013)	Prolonged mate guarding (Myers *et al*., 2015; Boldbaatar *et al*., 2024) Female resistance or avoidance (Schwander *et al*., 2013; Burke *et al*., 2015; Wilner, 2025) Parthenogens less attractive, have variable/modified chemical signals (Schwander *et al*., 2013; Burke *et al*., 2015; Ying *et al*., 2024; Wilner, 2025)	Yes (Larose *et al*., 2023; Schwander *et al*., 2013; Bedford, 1978; Morgan‐Richards *et al*., 2010, 2019; Wilner *et al*., 2025; Boldbaatar *et al*., 2025)	Mating: + Viability, + Fecundity (Bedford, 1978) cd Longevity, cd Fecundity, n Viability, cd Offspring survival, depending on previous reproductive mode (Burke *et al*., 2015) n Longevity, n Fecundity, + Viability, + Output (Burke & Bonduriansky, 2018a) + Fecundity, cd Viability, cd Output, depending on female impaternity (Burke & Bonduriansky, 2022) − Viability (some lineages; confounded with age) (Larose *et al*., 2023) cd Fecundity, cd Viability, cd Output, depending on female lineage and impaternity (Wilner, 2025) Rearing environment: − Viability, −Output (Burke & Bonduriansky, 2019a)	Mating: − Viability, − Offspring fertility (Marescalchi & Scali, 2003) n Viability (Schwander *et al*., 2013)
Opiliones	Yes	Enlarged claspers/pedipalps (but no sclerotised female pregenital barriers) in facultative species (Burns & Tsurusaki, 2016)		Unknown (but see Brown *et al*., 2021)	(Observational) Sex ratio: n Fecundity (Brown *et al*., 2021)	
Mayflies	Yes				Mating: + Hatching success (Liegeois *et al*., 2021)	Mating: − Hatching success (Liegeois *et al*., 2021)
*Drosophila*	Tychoparthenogenesis (artificially selected lines facultative)		Selection for parthenogenetic ability leads to reduced mating (Carson *et al*., 1982)		Mating: n Fecundity in lines selected for parthenogenesis (positive effect in control lines) (Crews *et al*., 1985)	
Cockroaches	Tychoparthenogenesis and closely related sexual and asexual lineages		Resistance to mating in females from asexually reproducing lineages (Roth & Willis, 1961)		Male odours: + Fertility (Moore & Moore, 2003)	Mating: − Fertiliity (Roth & Willis, 1961)
Brine shrimp	Unknown (all considered obligately sexual or asexual, but facultatively parthenogenetic hybrid individuals found)					(Observational, indirect evidence) Invasive sexual lineages displacing native parthenogens (Amat *et al*., 2005, 2007)
Ostracods	Unknown (a few suspected)		Females from asexually reproducing lineages less likely to copulate (Schmit *et al*., 2013)			
Lizards	Closely related sexual and asexual lineages		‘Copulation marks’ (scars from male bites) on wild parthenogens (Carretero *et al*., 2018) Male aggression (Bolger & Case, 1992; Galoyan *et al*., 2025)			(Observational, indirect evidence) A sexual species ecologically displaces parthenogens (Weterings & Vetter, 2018)

While the fitness effects of interactions with males appear to be very context dependent for facultatively parthenogenetic females, they seem to be consistently negative for obligate parthenogens (Table 2), suggesting that interactions with males from closely related sexual lineages could hamper the success of asexual lineages. For example, obligately asexual mayflies experience reduced hatching success if they mate (Liegeois *et al*., 2021), and obligately asexual cockroaches produce fewer offspring (Roth & Willis, 1961). This could be due to sperm or seminal fluids interfering with the development of parthenogenetic eggs (which might also occur in some facultative parthenogens; Burke & Bonduriansky, 2022) or otherwise harming eggs or females. Some evidence points to other factors as well, such as the cost of female resistance and harmful male behaviours. For example, female cockroaches from all‐female lineages have been observed resisting male mating attempts (Roth & Willis, 1961). Also, male bite marks are regularly found on obligately asexual female lizards (Carretero *et al*., 2018), and these females could be under selection to avoid mating with males from closely related sexual lineages because some of the hybrid offspring resulting from such copulations may have reduced fertility (although this has not been confirmed; Danielyan *et al*., 2008). Nonetheless, with so few relevant studies, it is not yet possible to draw firm conclusions beyond the need for more work on these animals.

Two main issues currently hinder our understanding of the role of sexual conflict in limiting or enhancing the evolution of parthenogenesis: the lack of knowledge of the basic life history and behaviour of most of the relevant organisms, and the lack of studies that incorporate a full picture of the fitness effects of sex (i.e. considering both immediate or direct effects on female fecundity, as well as more indirect effects on the reproductive success of offspring). Firstly, in order to assess hypotheses about the evolution of reproductive modes, we need a better understanding of which animals are capable of them, and to what extent they employ them. In some cases, animals are considered obligately parthenogenetic because males have not been described, and many other species are considered facultatively parthenogenetic because some populations have males and others do not. But breeding experiments are necessary to confirm whether (and to what extent) the animals in question are able to reproduce sexually and parthenogenetically when given the chance, and screening in wild populations is necessary to determine to what extent they do so in the wild. All‐female populations do not necessarily stem from obligate parthenogenesis, just as an equal sex ratio does not necessarily mean that the females in a population are obligately sexual. Many putative obligately sexual animals may in fact be tychoparthenogenetic, or even facultatively parthenogenetic because, usually, the ability to reproduce parthenogenetically is not easily observed without testing for it experimentally (Markow, 2013; Schwander *et al*., 2010). Sex ratio is often used as an indicator of parthenogenetic ability, but low levels of parthenogenesis can occur without substantially biasing population sex ratios, and some facultative parthenogens can form populations that have near‐even sex ratios (e.g. *Megacrania batesii*; Miller *et al*., 2024a). Further, in some systems where males can be produced asexually (e.g. animals with ZZ/ZW or XX/XO sex‐determination), asexual reproduction may not lead to biased sex ratios at all. Therefore, many more species may be facultatively parthenogenetic or tychoparthenogenetic than we currently have accounted for, as suggested by recent work on flies (Markow, 2013; Sperling & Glover, 2023). Even species that are well studied for other purposes sometimes have unknown or uncertain reproductive modes because there has been little or no experimental research on their reproductive biology. We hope this review will inspire readers to look more closely at their study animals' reproductive habits.

Further difficulties arise from information that is known but not readily available or clearly discussed. Many articles fail to mention or explain the reproductive biology of their study systems, even when it has been previously studied. Usually, such articles are focused on a different aspect of the animals' biology, but even so, the reproductive mode of the animal in question could have important implications for the interpretation of the study's results. Many articles simply refer to animals as ‘asexual’ or ‘parthenogenetic’, failing to clarify whether the organisms in question belong to obligately, facultatively, or cyclically asexual lineages or species (or whether this information is unknown). Interpretation of existing evidence is also complicated by disagreements over terminology. For example, many authors use the term ‘facultative parthenogenesis’ loosely to include cyclical parthenogenesis and anecdotal or rare parthenogenesis events, as well as truly facultative parthenogenesis, where each individual female is capable of both sexual and asexual reproduction. More databases like the one recently published for mayflies (Liegeois *et al*., 2020) would be extremely useful.

Perhaps most importantly, very few studies on parthenogenetic animals have investigated behaviour and the fitness effects of intersexual interactions (within or between species), and especially few studies have investigated the role of sexual conflict in such systems. Morphology and behaviour are sometimes studied, but often separately. To push the field forward, we will need to connect these different aspects (e.g. Brown *et al*., 2025), and tie them to their fitness consequences. Several studies have compared the reproductive output of mated and unmated parthenogens over a limited period of time (e.g. in stick insects), but effects on other components of fitness – such as female longevity, risk of predation and infection, and offspring reproductive performance, including that of sons – have rarely been considered. Studies examining the relative costs and benefits of sex and parthenogenesis for facultative females under different contexts (environmental, social, genetic) are even more rare. We will especially need more work examining fitness outcomes in ecologically relevant contexts and in natural populations, so that we can understand how effects obtained from laboratory experiments translate to the wild.

Overall, there is evidence of sexually antagonistic interactions in taxa with parthenogenesis, as well as interactions that may prevent or hinder parthenogenesis, but there is much work ahead. Our review of the literature shows that despite the abundance of relatively well‐studied parthenogens, there is a surprising lack of research into their behaviour. Little to nothing is known about the sexual behaviour of most facultative species, or about the interactions between obligate asexuals and the males of closely related sexual species. Very often it is not even clear whether putative asexuals are obligate or facultative (although, admittedly, it is no easy task to determine this; Schurko, Neiman & Logsdon, 2009). Several model parthenogens are already being studied across animal taxa, from vertebrates to invertebrates, but we have just started to scratch the surface when it comes to their behaviour and life history. Given the potential for evidence on this topic to shed light on the paradox of sex (Burke & Bonduriansky, 2017; Kawatsu, 2013a), research in the area is likely to be rewarding.

What behavioural and morphological evidence exists is largely consistent with predictions of the sexual conflict hypothesis. Putatively sexually antagonistic morphological traits have been described in facultative and obligately asexual stick insects (Schwander *et al*., 2013), and in males (but not females) of facultative Opiliones species (Burns & Tsurusaki, 2016). There is also evidence of male sexual behaviours that may be costly to parthenogens, such as pre‐mating struggles (Burke *et al*., 2015) and prolonged mate guarding in stick insects (Myers *et al*., 2015; Boldbaatar *et al*., 2024), and male lizards injuring parthenogens during mating attempts and struggles (Carretero *et al*., 2018; Galoyan *et al*., 2025). Additionally, the evolution of parthenogenesis frequently appears tied to females exhibiting increased chemical and/or behavioural avoidance or resistance to mating (Schwander *et al*., 2013; van der Kooi & Schwander, 2014; Burke *et al*., 2015; Ying *et al*., 2024; Wilner, 2025; Carson *et al*., 1982; Roth & Willis, 1961) and/or fertilisation (Wilner *et al*., 2025; Schwander *et al*., 2013; Larose *et al*., 2023; Bedford, 1978; Morgan‐Richards *et al*., 2010, 2019).

However, very few empirical studies have explicitly set out to test the sexual conflict hypothesis, and much of the available evidence is incidental. Additionally, the fitness effects of the above traits and interactions are poorly understood and largely unstudied. Available evidence suggests that mating is generally costly for obligate parthenogens (Marescalchi & Scali, 2003; Liegeois *et al*., 2021; Roth & Willis, 1961), but effects on facultative parthenogens appear much more complex and context dependent (see Table 2). This is a particularly important gap in knowledge because sexually antagonistic traits can also evolve through mate choice rather than sexual conflict, and may not always result in conflicting fitness effects between the sexes (Cordero & Eberhard, 2003). Rigorous study of fitness consequences is thus necessary to differentiate between mate choice and sexual conflict, particularly since direct and indirect costs and benefits may counteract each other.

Moreover, some traits that facilitate female resistance or avoidance, such as physiological barriers to fertilisation (e.g. loss of sperm storage organs) or the loss of male‐attracting pheromones, may also evolve through vestigialisation, due to neutral decay processes (e.g. genetic drift and accumulation of mutations in traits that are now under relaxed selection), or natural selection for reduced expression of traits that are energetically costly or subject to trade‐offs with more beneficial traits (van der Kooi & Schwander, 2014). Distinguishing between neutral and selection‐driven vestigialisation is challenging, and requires an understanding of the trait's fitness effects, correlations with other traits, and evolutionary history. It is worth noting however, that these are not mutually exclusive processes. Sexual conflict is likely to interact with other selection pressures, and their effects may be compounded. For example, sexual traits may be selected against both because they lead to costly interactions with males *and* because they are energetically costly to produce and maintain. Additionally, the outcomes of sexually antagonistic coevolution in facultatively parthenogenetic populations may set the scene for vestigialisation of sexual or parthenogenetic traits (see Fig. 1): if females rarely get the chance to reproduce parthenogenetically, selection on the necessary physiological systems may be relaxed; whereas if males disappear from a population, sexual traits will likely lose their usefulness.

The evidence we do have suggests that sexual interactions between males and parthenogenetic females occur frequently, both within and between species, and that many of these interactions might be costly to females and therefore antagonistic. As predicted by theory (Kawatsu, 2015; Burke & Bonduriansky, 2019b), the evidence also suggests that many of these interactions have the potential to hinder the evolution of parthenogenesis, by preventing females from reproducing parthenogenetically, or by hindering their success when they do. Conversely, the evolution of effective female resistance could enable facultative strategies to invade sexual populations.

## IV. CONCLUSIONS

(1) Morphological, physiological, and behavioural traits suggest a history of sexual conflict in several facultatively parthenogenetic systems, but much remains unknown.
(2) Available fitness data suggest that interactions with males tend to impact obligate parthenogens negatively, but in facultative systems, the relative costs and benefits of sex and parthenogenesis appear to be highly context dependent.
(3) Life history and behaviour remain understudied and poorly understood in most parthenogens, and in many cases, even the reproductive mode is unknown or unclear.
(4) To evaluate the sexual conflict hypothesis properly and understand the evolution of parthenogenesis and sex, we will need studies that incorporate a full picture of the fitness effects of sexual reproduction and the traits it entails, under different environmental conditions, and in natural populations of facultative parthenogens.
